# ^1^H NMR Metabolomics and Full-Length RNA-Seq Reveal Effects of Acylated and Nonacylated Anthocyanins on Hepatic Metabolites and Gene Expression in Zucker Diabetic Fatty Rats

**DOI:** 10.1021/acs.jafc.1c00130

**Published:** 2021-04-09

**Authors:** Kang Chen, Xuetao Wei, Raghunath Pariyani, Maaria Kortesniemi, Yumei Zhang, Baoru Yang

**Affiliations:** † Food Chemistry and Food Development, Department of Life Technologies, University of Turku, FI-20014 Turun yliopisto, Finland; ‡ Beijing Key Laboratory of Toxicological Research and Risk Assessment for Food Safety, Department of Toxicology, School of Public Health, Beijing University, Beijing 100191, China; § Department of Nutrition and Food Hygiene, School of Public Health, Beijing University, Beijing 100191, China

**Keywords:** nonacylated anthocyanins, acylated anthocyanins, nontargeted ^1^H NMR metabolomics, full-length RNA-Seq, type 2 diabetes

## Abstract

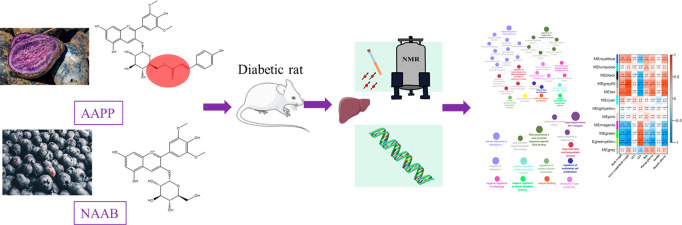

Anthocyanins have been reported to possess antidiabetic effects. Recent studies indicate acylated anthocyanins have better stability and antioxidative activity compared to their nonacylated counterparts. This study compared the effects of nonacylated and acylated anthocyanins on hepatic gene expression and metabolic profile in diabetic rats, using full-length transcriptomics and ^1^H NMR metabolomics. Zucker diabetic fatty (ZDF) rats were fed with nonacylated anthocyanin extract from bilberries (NAAB) or acylated anthocyanin extract from purple potatoes (AAPP) at daily doses of 25 and 50 mg/kg body weight for 8 weeks. Both anthocyanin extracts restored the levels of multiple metabolites (glucose, lactate, alanine, and pyruvate) and expression of genes (*G6pac*, *Pck1*, *Pklr*, and *Gck*) involved in glycolysis and gluconeogenesis. AAPP decreased the hepatic glutamine level. NAAB regulated the expression of *Mgat4a*, *Gstm6*, and *Lpl*, whereas AAPP modified the expression of *Mgat4a*, *Jun*, *Fos*, and *Egr1*. This study indicated different effects of AAPP and NAAB on the hepatic transcriptomic and metabolic profiles of diabetic rats.

## Introduction

Anthocyanins, a major class of polyphenolic compounds in plants, are known to have a modulatory effect on oxidative stress, inflammation, and energy homeostasis.^1^ The core structure of anthocyanins is anthocyanidin (Figure S1A) which can be glycosylated to form nonacylated anthocyanins (Figure S1B), and the glycosides can be acylated to form acylated anthocyanins (Figure S1C).^2^ The acylation process of anthocyanins is regulated by acyltransferase in plants.^3^ Compared to the nonacylated anthocyanins, acylated anthocyanins are more stable in the environments of varying pH, heat, and light,^4^ and they have been reported to have higher antioxidant activity,^5^ likely due to the stacking of the acyl groups with the pyrylium ring reducing the susceptibility of nucleophile attack of water and increasing the stability.^6^

Although anthocyanins and other phenolic compounds have been shown to have positive effects in regulating sugar metabolism and reducing the risk of type 2 diabetes (T2D),^7^ very little evidence is available showing the impacts of these compounds on the overall metabolomics profile of the healthy, prediabetic or diabetic state. In our previous study using an NMR-metabolomics method, nonacylated and acylated anthocyanins displayed a different beneficial effect on the plasma metabolic profile of ZDF rats.^8^ The liver plays a crucial role in the pathogenesis of T2D by regulating glucose metabolism and metabolic homeostasis.^9^ We hypothesize that the anthocyanins regulate the hepatic metabolic profile by regulating the expression of the genes involved in energy metabolism in the liver; these changes can be revealed most efficiently by the powerful combination of high throughput methods of metabolomics and transcriptomics. Further, we hypothesize that acylated anthocyanins and nonacylated anthocyanins have different effects on energy metabolism in the state of type 2 diabetes *in vivo* due to the difference in solubility, stability, and bioavailability.

Metabolomics and transcriptomics have been demonstrated to be powerful approaches to reveal the changes in the complicated regulatory networks of metabolic pathways.^10^ Metabolomic analysis revealed that anthocyanins extract from raspberry recovered hepatic metabolites involved in glutathione metabolism, the insulin signaling pathway, and glycerophospholipid metabolism in obese mice.^11^ Transcriptomics has illustrated that pelargonidin-3-*O*-glucoside extracted from raspberries could modulate hepatic gene expression associated with glucose and lipid metabolism in *db/db* mice.^12^

Recent advances in nanopore sequencing technology allow sequencing of long RNA sequences up to 100 kb, which spans the full-length distribution of spliced genes in humans.^13^ Furthermore, nanopore sequencing has proven to be even more sensitive compared to real-time PCR in detecting bacterial biodefense pathogens.^14^ Detecting full-length mRNA for accurate quantification of alternative transcripts and genes is crucial for understanding the molecular pathways disrupted in disease progression and the possible regulating effects of food and diet on these pathways.

In this study, our objective was to investigate the potential different impact of nonacylated anthocyanins extracted from bilberries (NAAB) and acylated anthocyanins extracted from purple potatoes (AAPP) on the hepatic metabolism of ZDF rats as an animal model of T2D. Changes of hepatic metabolites affected by anthocyanin extracts in ZDF rats were detected by using ^1^H NMR-based metabolomics, and alterations in gene expression were studied using full-length RNA-Seq transcriptomics. To the best of our knowledge, this is the first study where an integrated application of these two powerful techniques has been applied to study changes in hepatic gene expression and metabolites. Our findings form new insights on the impacts of nonacylated and acylated anthocyanins on the diabetic state and the molecular mechanisms responsible for the observed metabolic and transcriptomic changes.

## Materials and Methods

### Animals and Diets

Male ZDF rats (*fa*/*fa*) fed with high-fat diet Purina #5008 were used as a model of type 2 diabetes.^15^ Lean Zucker rats (*fa/+*) were used as healthy control. Nonacylated anthocyanins were extracted from bilberry (*Vaccinium myrtillus* L.) and acylated anthocyanins from purple potato (*Solanum tuberosum* L.) of the cultivar “Synkeä Sakari”. Detailed methods on the extraction, identification, and compositional analysis of anthocyanins were described in our previous study,^8^ anthocyanins in bilberry were only found nonacylated, whereas 98.97% of anthocyanins in purple potato were acylated.^8^

Rat housing and group designation were described in a previous study.^8^ Briefly, 40 ZDF rats (*fa*/*fa*) were divided into five groups: ZDF rats fed with high-fat Purina #5008 diet as the diabetic model group (M); ZDF rats fed with Purina #5008 diet and gavaged with a low dose of nonacylated anthocyanin from bilberry (25 mg/kg body weight/day, L-NAAB); ZDF rats fed with Purina #5008 diet and gavaged with a low dose of acylated anthocyanin from potato (25 mg/kg body weight/day, L-AAPP); ZDF rats fed with Purina #5008 diet and gavaged with a high dose of nonacylated anthocyanin from bilberry (50 mg/kg body weight/day, H-NAAB); ZDF rats fed with Purina #5008 diet and gavaged with a high dose of acylated anthocyanin from potato (50 mg/kg body weight/day, H-AAPP). Sixteen of the lean Zucker rats (*fa*/*+*) were divided into two groups, one fed with normal diet (ND) and the other with Purina #5008 diet (Con). M and Con groups are both on a high-fat diet Purina #5008, and comparison of these two groups reveals the impact of *leptin* receptor gene deficiency as the single variable differing between the two groups. Lean Zucker rats fed with a normal diet (ND) represent the normal metabolic profile of the healthy state. The compositions of the normal diet and the Purina #5008 diet were listed in our previous study.^8^ Each group contained 8 rats. The dosages of anthocyanin extracts were determined based on previous animal^16^ and human^17^ studies. After 8 weeks of intervention, the rats were fasted for 12 h and sacrificed under isoflurane anesthesia. Mass of liver, epididymal fat, as well as their percentages of body weight were measured. The livers were frozen immediately in liquid nitrogen and stored at −80 °C.

### Liver RNA Extraction and cDNA Preparation

Total RNA was extracted from the liver tissue using TRIzol reagent (Takara, Kyoto, Japan) according to the manufacturer’s instructions. RNA purity was tested using the Nano Photometer spectrophotometer (IMPLEN, Westlake Village, USA). cDNA libraries were constructed from 1 μg of total RNA by using a cDNA-PCR Sequencing Kit (SQK-PCS109) according to the protocol. Briefly, the reverse transcriptase was used to enrich full-length cDNAs and added defined PCR adapters to both ends of the first-strand of cDNA and following cDNA PCR for 14 circles with LongAmp Tag DNA polymerase (New England Biolabs, Ipswich, MA, USA) (8 min for elongation time). The PCR products were then subjected to ONT adaptor ligation using T4 DNA ligase (New England Biolabs, Ipswich, MA, USA). Agencourt XP beads (Beckman Coulter, Brea, USA) were used for DNA purification. The final cDNA libraries were subjected to FLO-MIN109 flow cells and analyzed on a PromethION platform at Biomarker Technology Company (Beijing, China).

### Transcriptome Data Analysis

First, raw reads with average read quality score below 7 and read length below 500 bp were filtered out. After mapping to the rRNA database, the rRNA was discarded. Next, full-length, nonchimeric (FLNC) transcripts were identified by searching for primers at both ends of the reads. The Mimimap2 alignment program^18^ was used to obtain clusters of FLNC transcripts by mapping to the reference genome library. The consensus isoforms were obtained after polishing within each cluster by pinfish (https://github.com/nanoporetech/pinfish). Mapped reads were further collapsed by the *cDNA_Cupcake* package (https://github.com/Magdoll/cDNA_Cupcake) with min-coverage = 85% and min-identity = 90%. Full-length reads were mapped to the reference genome (Rnor_5.0). Reads with match quality over 5 were further used for quantification. Expression levels were estimated by CPM, counts per million. CPM = number of reads mapped to transcript/total reads aligned in sample ×1,000,000. Package *edgeR* was used for differential expression analysis of two groups.^19^ The resulting p values were adjusted by Benjamini and Hochberg’s approach. Genes and transcripts with a p value < 0.05 and fold change ≥ 2 were considered as differentially expressed genes (DEGs) and differentially expressed transcripts (DETs).

### Protein–protein Interaction (PPI) Network Construction

A protein–protein interaction (PPI) network can be used to map networks of protein interactions depending on their physical or functional association. It represents a platform by which it is possible to systematically identify disease-related genes.^20^ DEGs were used for network construction based on protein–protein interaction by the Search Tool for the Retrieval of Interacting Genes/Proteins (STRING; https://string-db.org/). The parameters of the gene interactions generated from STRING were imported into Cytoscape (Version:3.2.1; https://cytoscape.org/) to form a PPI network and to identify the hub genes with high degree. Cytoscape is a software that is capable of integrating data into a unified conceptual framework. The parameter of the degree measures how many neighbors a node directly connects to. In this study, the top 10 genes with the highest degree were considered as hub genes.

### Enrichment Analysis Based on KEGG and GO

KEGG (Kyoto Encyclopedia of Genes and Genomes) and GO (Gene Ontology) are commonly used bioinformatic libraries that provide comprehensive information on the functions of genes. The KEGG pathway enrichment analysis of DEGs was implemented using the *enrichKEGG* function based on R software (Version 3.4.1). In KEGG pathway enrichment analysis, those KEGG pathways with q values less than 0.25 and rich factor greater than 1 were considered significantly enriched. The greater the rich factor, the higher the degree of enrichment. For functional network visualization, ClueGO visualizes the selected GO terms in a functionally grouped annotation network that reflects the relationships between the terms based on the similarity of their associated genes.^21^ The size of the nodes indicates the statistical significance of the terms. The degree of connectivity between terms (edges) is calculated using kappa statistics. Only significantly enriched GO terms with kappa score greater than 0.03 were shown in the network.

### Extraction of Hepatic Metabolites for ^1^H NMR-Based Metabolomics Assays

The liver was homogenized in liquid nitrogen, and around 0.2 g of homogenized liver sample was weighed and suspended in methanol (4 mL g^–1^) and Milli Q water (0.85 mL g^–1^). After vortexing, 2 mL g^–1^ of chloroform was added followed by vortexing again. Then, chloroform (2 mL g^–1^) and water (2 mL g^–1^) were added, and the mixture was vortexed for 1 min. Samples were left on ice for 30 min and then centrifuged at 2,000*g* for 10 min at 4 °C. The upper layer containing aqueous extract and the lower layer consisting of lipid extract were collected separately. Nitrogen gas and freeze-drying were used to remove the solvents from the lipid and aqueous extracts, respectively.

### NMR Spectroscopic Analysis

530 μL of phosphate buffer (90 mmol/L KH_2_PO_4_, pH = 7.4) and 70 μL of Chenomx Internal Standard (Chenomx Inc., Edmonton, Alberta, Canada) containing 5 mM DSS-*d*_6_ were added to the dried aqueous extract. The dried lipid extract was dissolved in 600 μL of chloroform-*d* containing 0.03% trimethylsilane (TMS). The solutions were transferred into 5 mm NMR tubes. The NMR acquisitions were performed at 298 K on a 600 MHz Bruker Avance-III NMR spectrometer (Bruker BioSpin AG, Fällanden, Switzerland) equipped with a Prodigy TCI cryoprobe and a precooled SampleJet sample changer. One-dimensional ^1^H NMR spectra were recorded from both liver extracts using the noesypr1d. The parameters for the noesypr1d pulse sequence were as follows: spectral sweep width, 16.02 ppm; data points, 64 K; total relaxation delay (RD), 5 s; acquisition time, 3.40 s; number of scans, 128. Phase and baseline correction were performed in the Chenomx Profiler 7.5 software (Chenomx Inc., Edmonton, Alberta, Canada). The chemical shift of DSS-*d*_6_ (δ = 0.00 ppm) or TMS (δ = 0.00 ppm) was used to align the spectra.

### Metabolite Identification

The NMR signal assignment and metabolite quantification were done with the aid of Chenomx Profiler 7.5 software (Chenomx Inc., Edmonton, Alberta, Canada), literature,^22,23^ and the Human Metabolome Database (HMDB, http://www.hmdb.ca). 2D NMR ^1^H–^13^C heteronuclear single-quantum correlation spectroscopy (HSQC) and *J*-resolved spectroscopy (JRES) were used to further confirm assignments. The 1D ^1^H NMR spectra of the hepatic lipid extract were binned into 0.01 ppm integrated spectral buckets. The 1D ^1^H NMR spectra of the hepatic aqueous extract were binned into 0.005 ppm integrated spectral buckets. The relative concentration of the metabolite was determined based on the binned integrated spectral buckets. Bins used for relative quantification are listed in Table S3 and Table S4.

### Weighted Gene Coexpression Network Analysis

Weighted gene coexpression network analysis (WGCNA) was used for the scale-free network topology analysis of RNA-Seq data. It was implemented using the WCGNA function based on R software (Version 3.4.1). Genes with similar expression patterns were classified into a module by calculating the expression correlation between genes. To broadly identify the coexpressed genes, all genes were used for analysis. To identify those modules with clinical relevance, Pearson’s correlation was used to analyze the correlation between modules and clinical parameters.

### Statistical Analysis

A two-side hypergeometric test was used as the statistical test method, and Benjamini–Hochberg was used as the FDR correction method in the GO/KEGG enrichment analysis. Data from ^1^H NMR metabolomics were tested for homogeneity of variances and normality by the Levene’s test and Kolmogorov–Smirnov test, respectively. One-way ANOVA was followed by the post hoc Bonferroni test when the data is normally distributed and variances were homogeneous or else the Kruskal–Wallis test and the post hoc Tamhane test were applied in SPSS statistics 22. The statistical significances are expressed as * *p* < 0.05, ** *p* < 0.01, and *** *p* < 0.001 compared to the M group.

## Result

### Liver, Epididymal Fat Weight, and General Observations of DETs and DEGs in Different Groups

**Figure 1:**
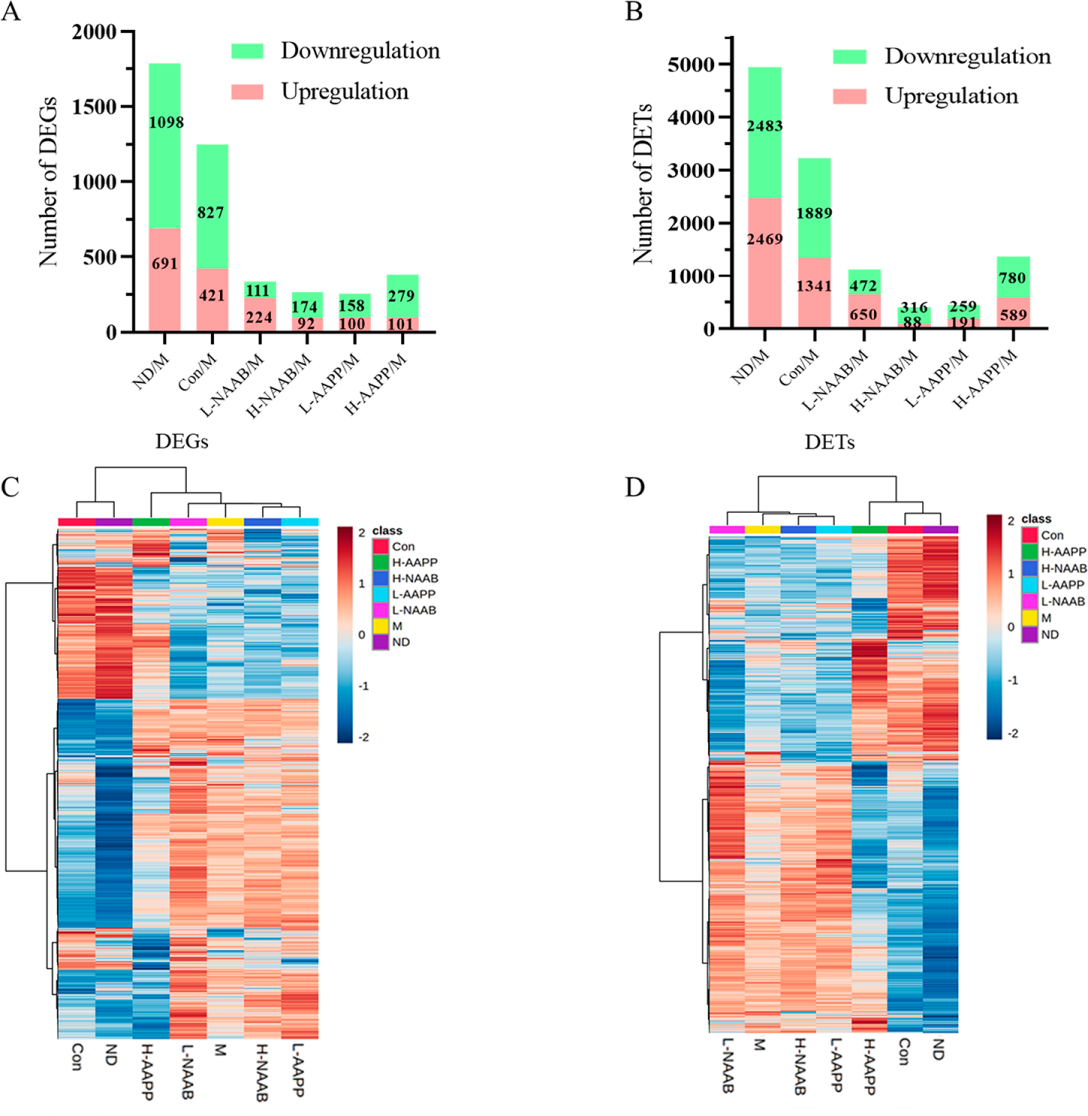
Statistics of DEGs (A) and DETs (B). Heatmap of DEGs (C) and DETs (D). In the heatmaps, red represents the upregulated genes or transcripts, blue represents the downregulated genes or transcripts. H, high dose; L, low dose; NAAB, nonacylated anthocyanins extracted from bilberries; AAPP, acylated anthocyanins extracted from purple potatoes.

The weight of liver and epididymal fat and their percentage of body weight in the M group were significantly higher than the corresponding values in the Con and ND groups (*p* < 0.05) (Table 1). Liver weight and the liver/body weight ratio of the Con group were higher than those in the ND group (*p* < 0.05). In the H-AAPP group, a significant decrease in the liver/body weight ratio was seen compared to the M group (*p* < 0.05). To understand the molecular mechanisms that anthocyanin extracts affected in T2D, we performed transcriptome profiling of the liver in the seven groups. Bioinformatics and statistical approaches were used to analyze the full-length RNA-Seq data. DEGs and DETs were identified in six comparisons between different groups: ND/M, Con/M, L-NAAB/M, H-NAAB/M, L-AAPP/M, and H-AAPP/M (Figure 1A and B). In the ND/M and Con/M comparisons, a large number of DEGs and DETs illustrated the distinguished transcriptome profiles caused by the difference between healthy control and the diabetic model (ND/M: 691 upregulated and 1098 downregulated DEGs, 2469 upregulated and 2483 downregulated DETs) and by deficiency in the *leptin* receptor gene (Con/M: 421 upregulated and 827 downregulated DEGs, 1341 upregulated and 1889 downregulated DETs). Among the anthocyanin extract treatment groups, the treatment with H-AAPP altered the largest numbers of genes and transcripts (101 upregulated and 179 downregulated DEGs, 89 upregulated and 780 downregulated DETs) compared to the M group. The heatmaps of the DEGs and DETs showed that the ND and Con groups were largely different from the M group in the pattern of gene expression; moreover, the transcriptome profile of the H-AAPP group appeared to be more similar to the ND and Con groups than the M group (Figure 1C and D).

**Table 1:** Effect of Anthocyanins from Bilberry and Purple Potato on Body Weight, The Mass of Liver, Epididymal Fat, and Their Percentage of Body Weight in Lean Zucker Rats (*fa/+*) and Obese Zucker Rats (*fa/fa*) Fed the Different Experimental Diets^a^

	Con	H-NAAB	H-AAPP	L-NAAB	L-AAPP	M	ND
Liver (g)	8.29 ± 0.98 b	14.67 ± 0.95 a	14.07 ± 2.30 a	14.40 ± 1.72 a	13.78 ± 0.73 a	14.67 ± 1.58 a	7.25 ± 0.88 c
Liver/body weight, %	4.34 ± 0.24 b	6.28 ± 0.37 a	6.13 ± 0.57 ab	6.30 ± 0.37 a	6.32 ± 0.37 a	6.61 ± 0.35 a	4.05 ± 0.14 c
Epididymal fat (g)	3.10 ± 0.38 b	7.95 ± 0.93 a	7.91 ± 1.20 a	7.81 ± 1.48 a	7.94 ± 0.65 a	7.57 ± 1.07 a	2.89 ± 0.70 b
Epididymal fat/body weight, %	1.62 ± 0.12 b	3.40 ± 0.21 a	3.44 ± 0.27 a	3.39 ± 0.22 a	3.64 ± 0.24 a	3.41 ± 0.32 a	1.60 ± 0.28 b

a Data represent the means ± SD. Values with different letters differ significantly in each row (*p* < 0.05). H, high dose; L, low dose; NAAB, nonacylated anthocyanins extracted from bilberries; AAPP, acylated anthocyanins extracted from purple potatoes (Figure 1).

### Genes Restored by Anthocyanin Extracts in ZDF Rats

**Figure 2:**
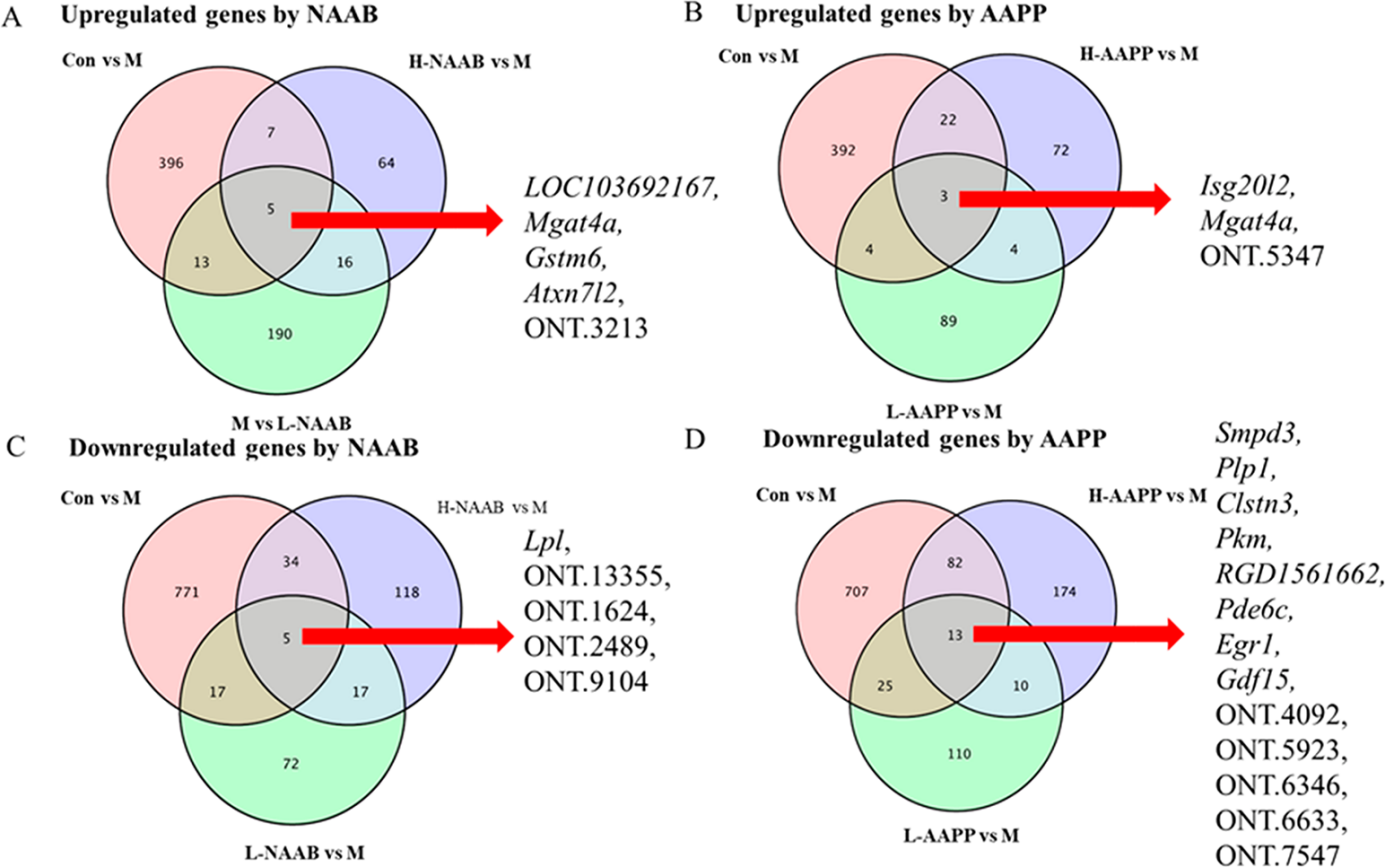
Venn diagrams showing downregulated genes in the M group compared to the Con group were upregulated by NAAB (A) and AAPP (B) and upregulated genes in the M group compared to the Con group were downregulated by NAAB (C) and AAPP (D). H, high dose; L, low dose; NAAB, nonacylated anthocyanins extracted from bilberries; AAPP, acylated anthocyanins extracted from purple potatoes.

Venn diagrams showed overlaps in upregulated and downregulated DEGs between comparisons, showing the genes restored by NAAB and AAPP in ZDF rats (Figure 2A–D), their annotation and involved pathways are shown in Table S1. The four known genes *LOC103692167, Mgat4a, Gstm6,* and *Atxn7l2*, and one unknown gene (labeled ONT.3213), of which the expression was decreased in the M group compared to the Con group, were upregulated by both L-NAAB and H-NAAB. The decrease in the expression of two genes *Isg20l2* and *Mgat4a* and one unknown gene (labeled ONT.5347) in the M group compared to the Con group was attenuated by L-AAPP and H-AAPP, shown as a higher level of expression of these genes in these groups than in the M group. The expression of one known gene *Lpl* and four unknown genes (labeled ONT.13355, ONT.1624, ONT.2489, and ONT.9104), which was increased in the M group compared to the Con group, was downregulated by both L-NAAB and H-NAAB. The L-AAPP and H-AAPP treatments downregulated the expression of eight known genes (*Smpd3, Plp1, Clstn3, Pkm, RGD1561662, Pde6c, Egr1,* and *Gdf15*) and five unknown genes (labeled ONT.4092, ONT.5923, ONT.6346, ONT.6633, and ONT.7547), which were increased in the M group compared to the Con group.

### PPI Network Construction

**Figure 3:**
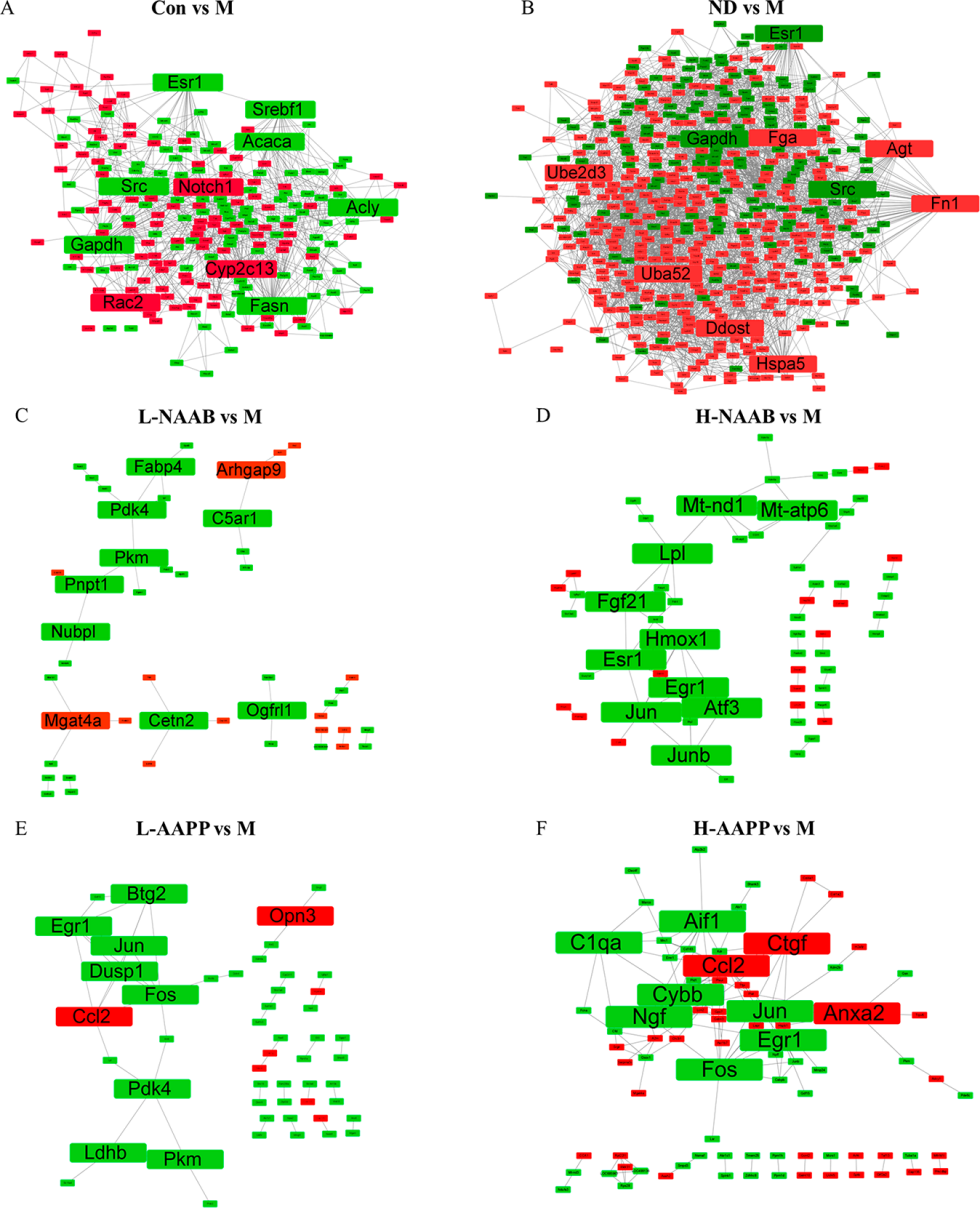
PPI network generated by DEGs in Con/M (A), ND/M (B), L-NAAB/M (C), H-NAAB/M (D), L-AAPP/M (E), and H-AAPP/M (F) comparisons. The top 10 genes with the highest degree were highlighted with bigger fonts. Genes with red color represent upregulation of genes in the group compared to the M group. Genes with green color represent downregulation of genes in the group compared to the M group. H, high dose; L, low dose; NAAB, nonacylated anthocyanins extracted from bilberries; AAPP, acylated anthocyanins extracted from purple potatoes.

We employed the PPI network construction based on DEGs in each comparison to show hub genes with high degree of connectivity in each comparison. The top 10 genes with the highest degree of connectivity with other genes were considered as hub genes (Figure 3A–F). The annotation of these genes and associated KEGG pathways are presented in Table S2. PPI network analysis revealed that *Gapdh, Src, Fasn, Notch1, Srebf1, Acly, Esr1, Rac2, Acaca*, and *Cyp2c13* were the top 10 genes with the highest degree of connectivity in the Con/M comparison. Hub genes recognized by PPI network analysis between healthy rats (ND) and diabetic rats (M) were *Fn1, Gapdh, Uba52, Src, Hspa5, Ddost, Agt, Fga, Ube2d3*, and *Esr1*.

The hub genes that were regulated by L-NAAB included *Pkm, Pdk4, Cetn2, Pnpt1, Fabp4, Mgat4a, C5ar1, Nubpl, Ogfrl1*, and *Arhgap9*, whereas the hub genes regulated by H-NAAB were *Jun, Esr1, Egr1, Hmox1, Atf3, Mt-nd1, Junb, Lpl, Fgf21,* and *Mt-atp6.* The potato anthocyanin extract regulated the hub genes *Fos, Egr1. Ccl2, Dusp1, Btg2, Jun, Pdk4, Pkm, Ldhb,* and *Opn3* at the low dose (L-AAPP) and *Ccl2, Fos, Jun, Ngf, Ctgf, Aif1, Cybb, Egr1, C1qa,* and *Anxa2* at the high dose (H-AAPP). Both *Jun* and *Egr1* were downregulated by H-NAAB, L-AAPP, and H-AAPP. *Fos* was downregulated by L-AAPP and H-AAPP.

### Functionally Grouped Annotation Network Based on ClueGO

**Figure 4:**
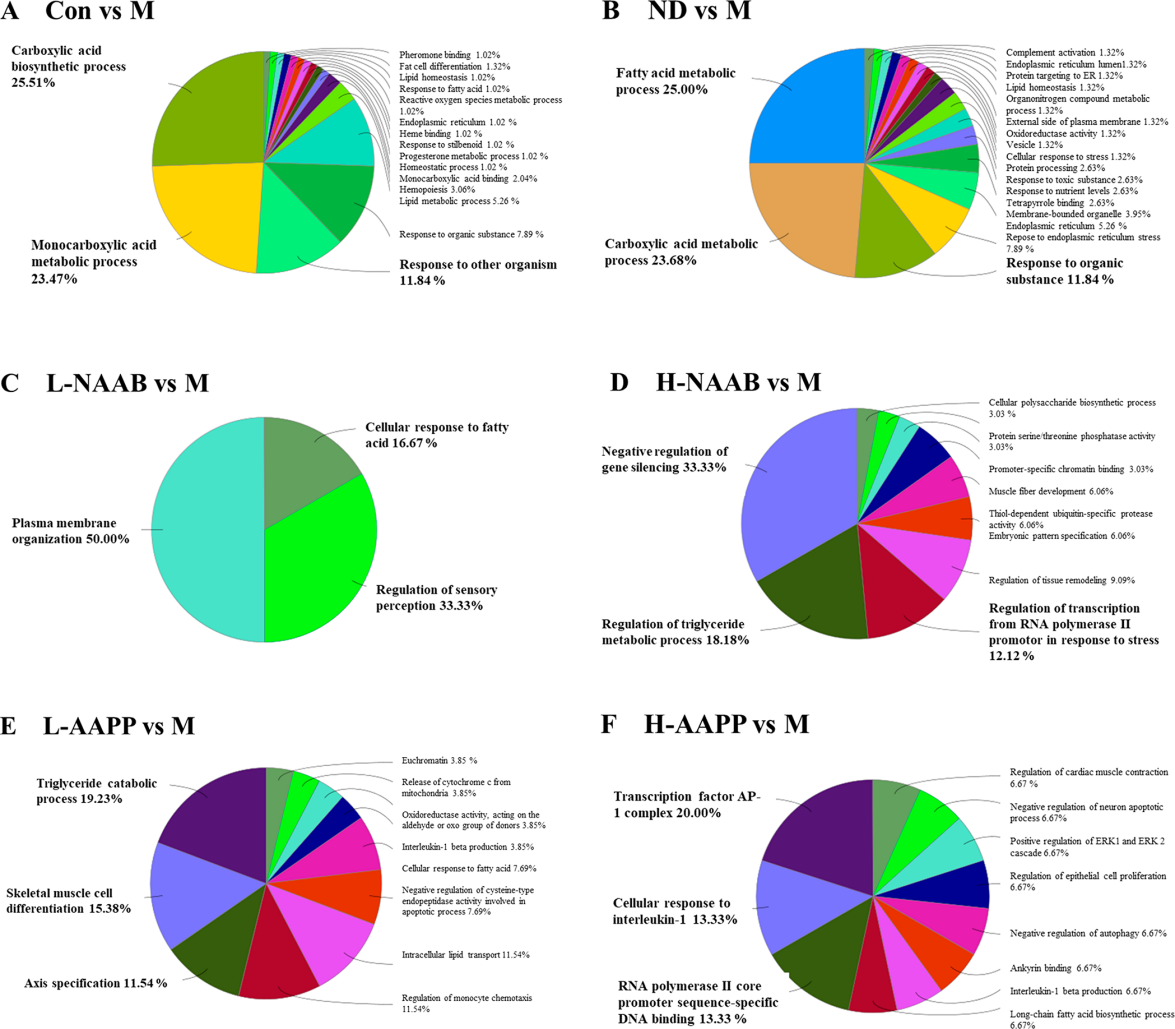
Overview chart generated from functionally grouped network of enriched GO terms based on DEGs in Con/M (A), ND/M (B), L-NAAB/M (C), H-NAAB/M (D), L-AAPP/M (E), and H-AAPP/M (F) comparisons. The top three functional groups with the most abundant GO terms in each comparison are highlighted in bold. H, high dose; L, low dose; NAAB, nonacylated anthocyanins extracted from bilberries; AAPP, acylated anthocyanins extracted from purple potatoes.

GO describes gene products associated biological processes, cellular components, and molecular functions. DEGs in each comparison were subjected to ClueGO to generate functionally grouped annotation networks (Figure S2A–F) and their overview charts presenting functional groups (Figure 4A–F). The top 3 functional groups with the most abundant GO terms in each comparison are highlighted in bold. The name of the functional group in the overview chart is given by the group leading term (the most significant term in the group). In the Con/M comparison, DEGs were mainly enriched in GO terms represented by the carboxylic acid biosynthetic process, the monocarboxylic acid metabolic process, and the response to other organisms. In the ND/M comparison, the fatty acid metabolic process, carboxylic acid metabolic process, and response to the organic substance represented the top 3 functional groups with the most abundant GO terms.

The networks generated by DEGs in ND/M and Con/M (Figure S2A and B) shared similar GO terms associated mainly with the monocarboxylic acid metabolic process, carboxylic acid biosynthetic process, fatty acid metabolic process, lipid metabolic process, and response to an organic substance. The network generated from DEGs in the L-NAAB/M comparison was associated with three functional groups: plasma membrane organization, regulation of sensory perception, and cellular response to fatty acids. In the H-NAAB/M comparison, DEGs were mainly enriched in negative regulation of gene silencing, regulation of the triglyceride metabolic process, and regulation of transcription from the RNA polymerase II promoter in response to stress. DEGs in the L-AAPP/M comparison were enriched in the triglyceride catabolic process, skeletal muscle cell differentiation, and axis specification. Transcription factor AP-1 complex, cellular response to interleukin-1, and RNA polymerase II core promoter sequence-specific DNA binding were found enriched in the H-AAPP/M comparison.

### KEGG Pathway Enrichment Analysis

**Figure 5:**
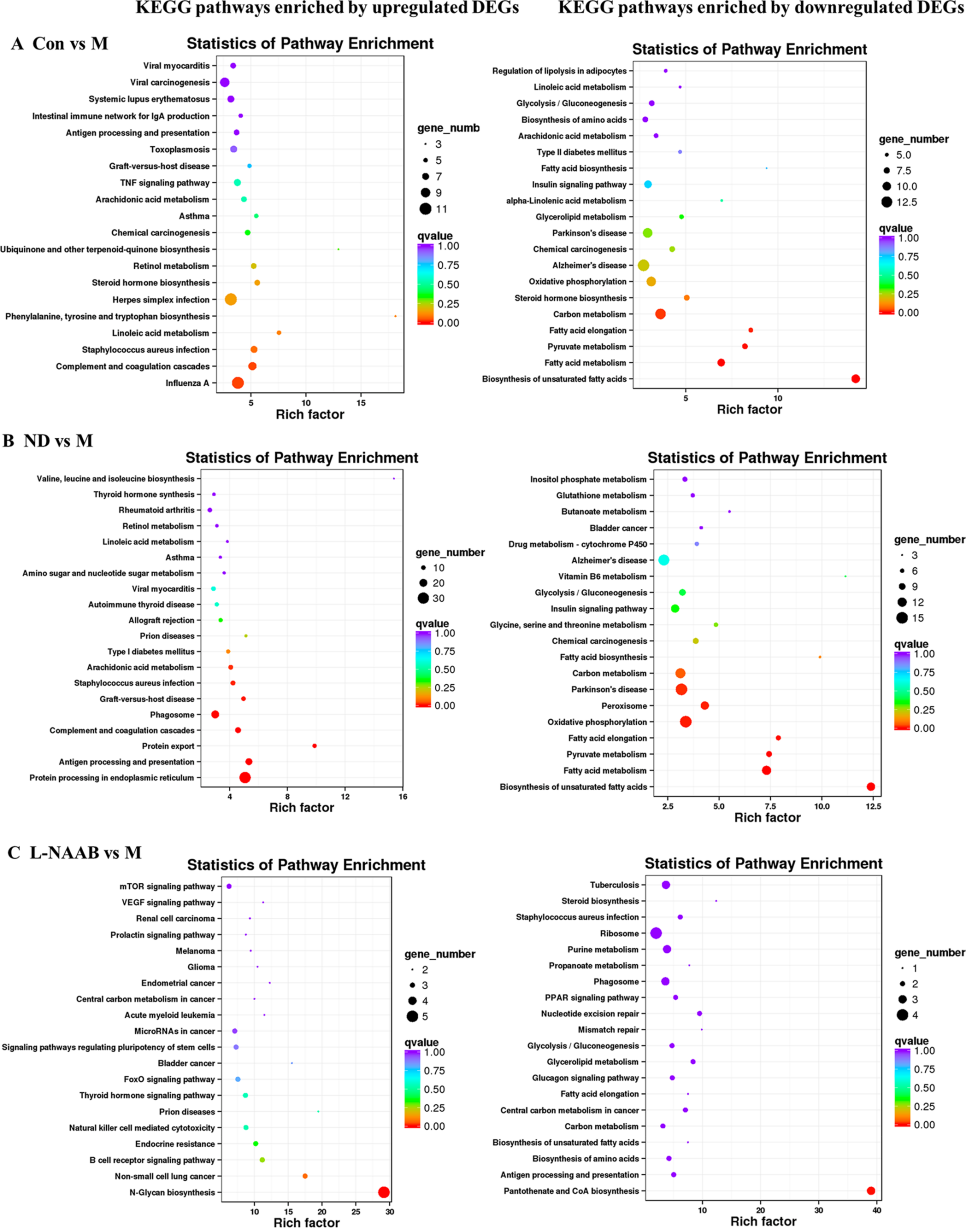

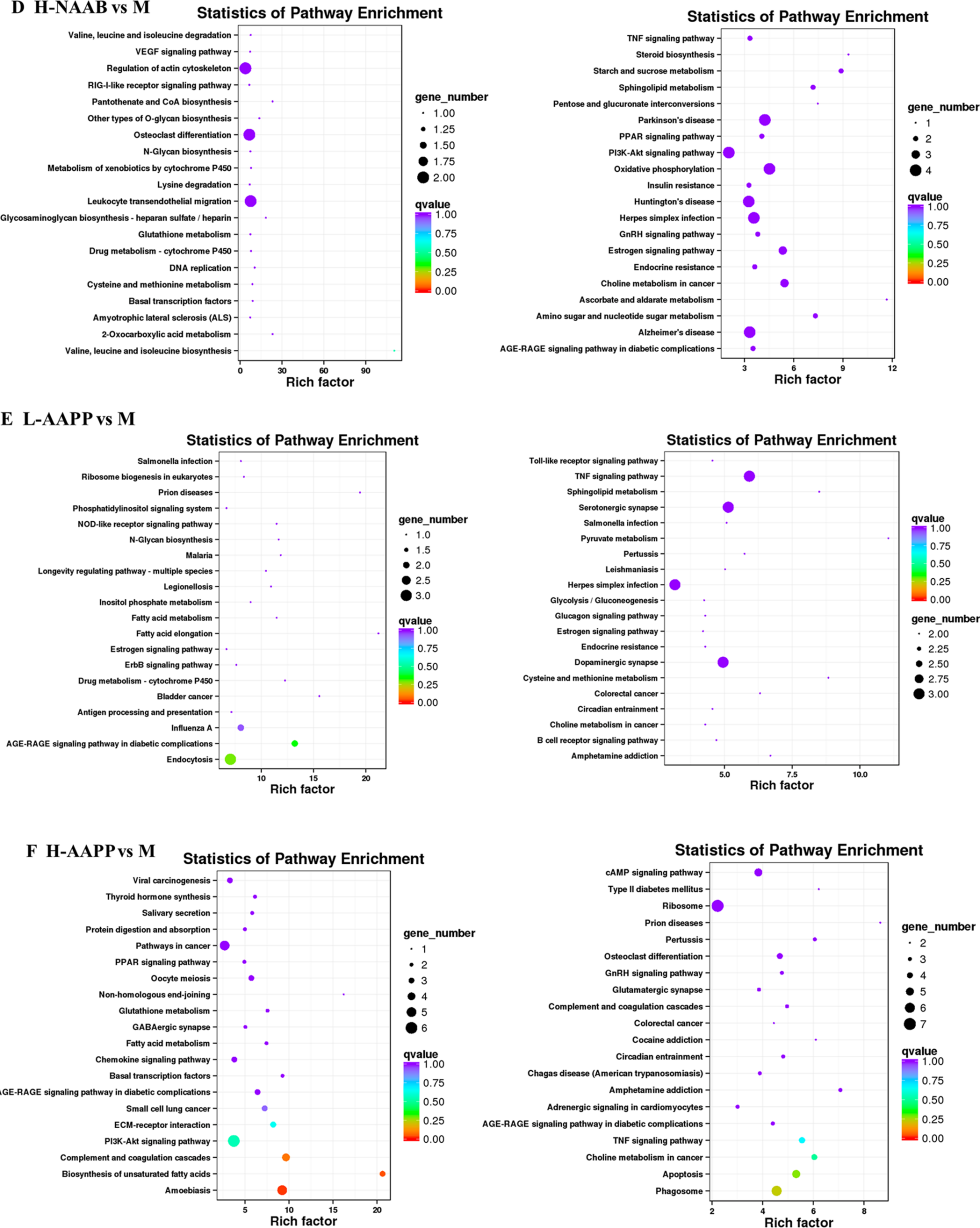
KEGG pathway enrichment analysis for upregulated genes (left panel) and downregulated genes (right panel) in Con/M (A), ND/M (B), L-NAAB/M (C), H-NAAB/M (D), L-AAPP/M (E), and H-AAPP/M (F) comparisons. The *y*-axis indicates the name of the KEGG pathway. The dot size means the gene number. The dot color indicates the q value. The top 20 KEGG pathways are presented. H, high dose; L, low dose; NAAB, nonacylated anthocyanins extracted from bilberries; AAPP, acylated anthocyanins extracted from purple potatoes.

Next, we performed KEGG pathway enrichment analysis. The top 20 pathways with the lowest q values were shown by a bubble chart (Figure 5A–F). Upregulated DEGs in the Con group compared to the M groups were mainly enriched in the following pathways: phenylalanine, tyrosine, and tryptophan biosynthesis, linoleic acid metabolism, *Staphylococcus aureus* infection, complement and coagulation cascades, and influenza A. Those upregulated DEGs in the ND group compared to the M groups were enriched in the pathways of arachidonic acid metabolism, *Staphylococcus aureus* infection, graft-versus-host disease, phagosome, complement and coagulation cascades, protein export, antigen processing and presentation, and protein processing in the endoplasmic reticulum.

Downregulated DEGs in Con/M and ND/M comparisons were enriched in similar KEGG pathways: biosynthesis of unsaturated fatty acid, fatty acid metabolism, pyruvate metabolism, fatty acid elongation, oxidative phosphorylation, and carbon metabolism.

For the anthocyanin treatment groups, upregulated DEGs in the L-NAAB group were significantly enriched in the N-glycan biosynthesis pathway. Downregulated DEGs in the L-NAAB group were significantly enriched in the pantothenate and CoA biosynthesis pathways. We did not see any KEGG pathways that were significantly enriched with downregulated DEGs in the H-NAAB, L-AAPP, and H-AAPP groups or with upregulated DEGs in the H-NAAB and L-AAPP groups. Upregulated DEGs in H-AAPP were significantly enriched in the pathways of complement and coagulation cascades, biosynthesis of unsaturated fatty acid, and amoebiasis.

### Metabolites Detected in ^1^NMR Metabolomics and Expression of Related Genes

We identified 38 metabolites from liver aqueous extracts and 17 from lipid extracts using ^1^H NMR metabolomics (Figure S3A–B). Identification was further aided by 2D NMR spectra (Figures S4–7 and Tables S3–4). To further investigate the alterations of the hepatic metabolites affected by the anthocyanin extracts, the fold change value and statistical significance compared to the M group were calculated and shown in Tables S5–6.

We found that the hepatic metabolic profiles differed between the M and the Con groups as well as between the M and ND groups. Compared to the lean Zucker rats (the Con and ND groups), the M group showed increased trends of glucose, lactate, alanine, pyruvate, leucine, isoleucine, valine, tyrosine, phenylalanine, glutamine, glutamate, glutamine/glutamate ratio, dimethylamine, dimethylglycine, choline, phosphocholine, taurine, maltose, glycine, glycerol, mannose, triglyceride, total phospholipid, fatty acids residue, polyunsaturated fatty acid, DHA, ARA and EPA, oleic acid, sphingomyelin, and monoglyceride (Tables S5–6).

**Figure 6:**
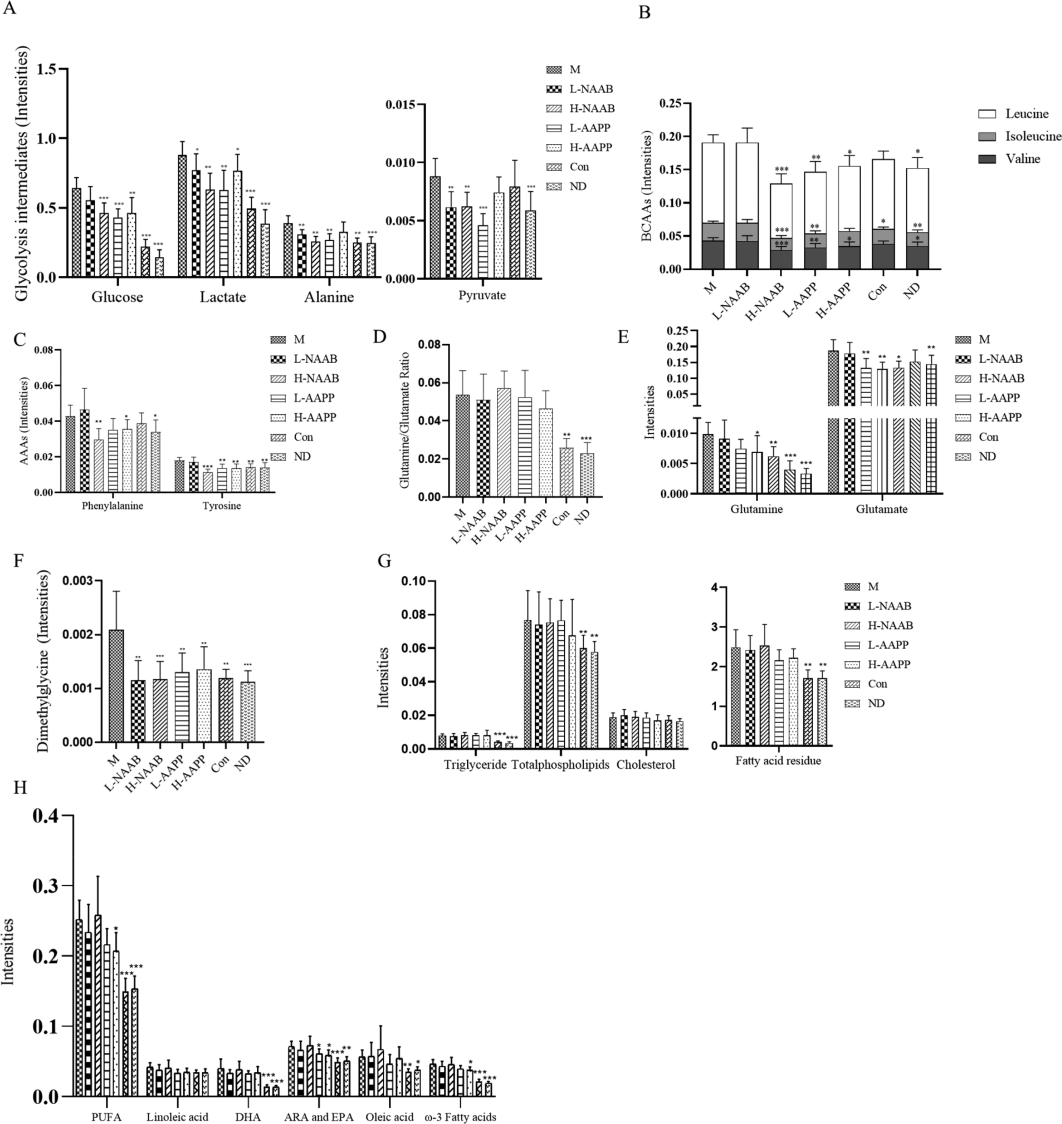
Effect of the supplement of L-NAAB, H-NAAB, L-AAPP, and H-AAPP on hepatic metabolites in ZDF rats. (A) Hepatic metabolites involved in glycolysis (glucose, lactate, alanine, and pyruvate). (B) Hepatic BCAAs levels (leucine, isoleucine, and valine). (C) Hepatic AAAs levels (aromatic amino acids: phenylalanine and tyrosine). (D) Glutamine/glutamate ratio. (E) Hepatic glutamine and glutamate levels. (F) Hepatic dimethylglycine level. (G) Hepatic triglyceride, total phospholipids, cholesterol, and fatty acid residues. (H) Hepatic unsaturated lipids. *: *p* < 0.05, **: *p* < 0.01, ***: *p* < 0.001 as compared with M group. H, high dose; L, low dose; NAAB, nonacylated anthocyanins extracted from bilberries; AAPP, acylated anthocyanins extracted from purple potatoes. FA, fatty acid; DHA, docosahexaenoic acid; EPA, eicosapentaenoic acid; ARA, arachidonic acid; PUFA, polyunsaturated fatty acids.

Treatment with the anthocyanin extracts from bilberry (H-NAAB, L-NAAB) and purple potato (H-AAPP and L-AAPP) led to changes in the levels of hepatic metabolites as compared to the M group, indicating a potential improvement of the diabetic state. Lower levels of metabolites involved in glycolysis (glucose, lactate, alanine, and pyruvate) were observed in all anthocyanin treatment groups (Figure 6A). L-NAAB, H-NAAB, L-AAPP, and H-AAPP groups decreased levels of glucose (*p* > 0.05, *p* < 0.001, *p* < 0.001, *p* < 0.01), lactate (*p* < 0.05, *p* < 0.01, *p* < 0.01, *p* < 0.05), alanine (*p* < 0.01, *p* < 0.01, *p* < 0.01, *p* > 0.05), and pyruvate (*p* < 0.01, *p* < 0.01, *p* < 0.001, *p* > 0.05).

We generated a heatmap for the expression of the genes in the glycolysis/gluconeogenesis pathway from the KEGG pathway library (Figure S8A). *Gck* and *Pklr, G6pc* and *Pck1* encode key enzymes in glycolysis and gluconeogenesis, respectively. *Gck* was found increased in the M group compared to the Con and ND groups (*p* < 0.01, *p* < 0.001), H-AAPP significantly reversed the increase in *Gck* expression (*p* < 0.05) while NAAB significantly increased *Gck* expression (L-NAAB vs M, *p* < 0.01; H-NAAB vs M, *p* < 0.05). Expression of *Pklr* was significantly higher in the M group compared to the Con and ND groups (*p* < 0.01, *p* < 0.001), L-AAPP and H-AAPP significantly decreased the expression of *Pklr* (*p* < 0.01, *p* < 0.01). We did not see differences in *Pck* and *G6pac* between the M group and Con and ND groups, while H-NAAB, L-AAPP, and H-AAPP significantly decreased *G6pac* (*p* < 0.05, *p* < 0.05, *p* < 0.05) and H-AAPP also significantly decreased *Pck1* (*p* < 0.05). Branched-chain amino acids (isoleucine, leucine, and valine) and aromatic amino acids (phenylalanine and tyrosine) were also affected by anthocyanin extracts (Figure 6B–C). Leucine and valine were significantly decreased by H-NAAB (*p* < 0.001, *p* < 0.001), L-AAPP (*p* < 0.01, *p* < 0.01), and H-AAPP (*p* < 0.05, *p* < 0.05). Isoleucine was decreased by H-NAAB (*p* < 0.001), and L-AAPP (*p* < 0.01). Phenylalanine as an essential amino acid was decreased by H-NAAB (*p* < 0.01) and H-AAPP (*p* < 0.05). Tyrosine was decreased by H-NAAB (*p* < 0.001), L-AAPP (*p* < 0.01), and H-AAPP (*p* < 0.01). However, *Pah* encoding phenylalanine hydroxylase responsible for the conversion of phenylalanine to tyrosine was not seen changed in the anthocyanin treatment groups compared to the M group (Figure S9A).

A higher glutamine/glutamate ratio in ZDF rats was not responsive to the anthocyanin extracts (Figure 6D); however, L-AAPP and H-AAPP decreased glutamine (*p* < 0.05, *p* < 0.01) and glutamate (*p* < 0.01, *p* < 0.05), and H-NAAB decreased glutamate (*p* < 0.01) (Figure 6E). *Gls2* and *Glul*, enabling interconversion of hepatic glutamine and glutamate, were not consistently changed with the hepatic glutamine and glutamate (Figure S9B–C).

In all the anthocyanin-fed groups the increased level of dimethylglycine was reversed toward the normal state (L-NAAB, *p* < 0.01; H-NAAB, *p* < 0.001; L-AAPP, *p* < 0.01; H-AAPP, *p* < 0.01, in comparison with M) (Figure 6F). *Bhmt* encoding betaine-homocysteine S-methyltransferase, responsible for catalyzing one step in the methionine cycle to produce dimethylglycine, was decreased by all treatment groups (L-NAAB, *P* > 0.05; H-NAAB, *p* > 0.05; L-AAPP, *p* < 0.05; H-AAPP, *p* < 0.05) compared to the M group (Figure S9D).

For the lipid metabolites, the L-AAPP and H-AAPP groups slightly improved the hepatic lipid profile shown as a decreasing trend in fatty acid residues (*p* > 0.05 and *p* > 0.05), unsaturated fatty acids including ω-3 fatty acids (*p* > 0.05 and *p* < 0.05) and ARA+EPA (*p* < 0.05 and *p* < 0.05) (Figure 6G–H). A heatmap of the expression of genes related to the biosynthesis of unsaturated fatty acids from the KEGG pathway library was generated (Figure S8B), the M group showed an overall upregulation of genes in this pathway, with *Acox1*, *Acox3*, and *Baat* being significantly decreased and *Pecr* significantly increased in the H-AAPP group (*p* < 0.05, *p* < 0.01, *p* < 0.05, and *p* < 0.05).

### WGCNA Analysis

**Figure 7:**
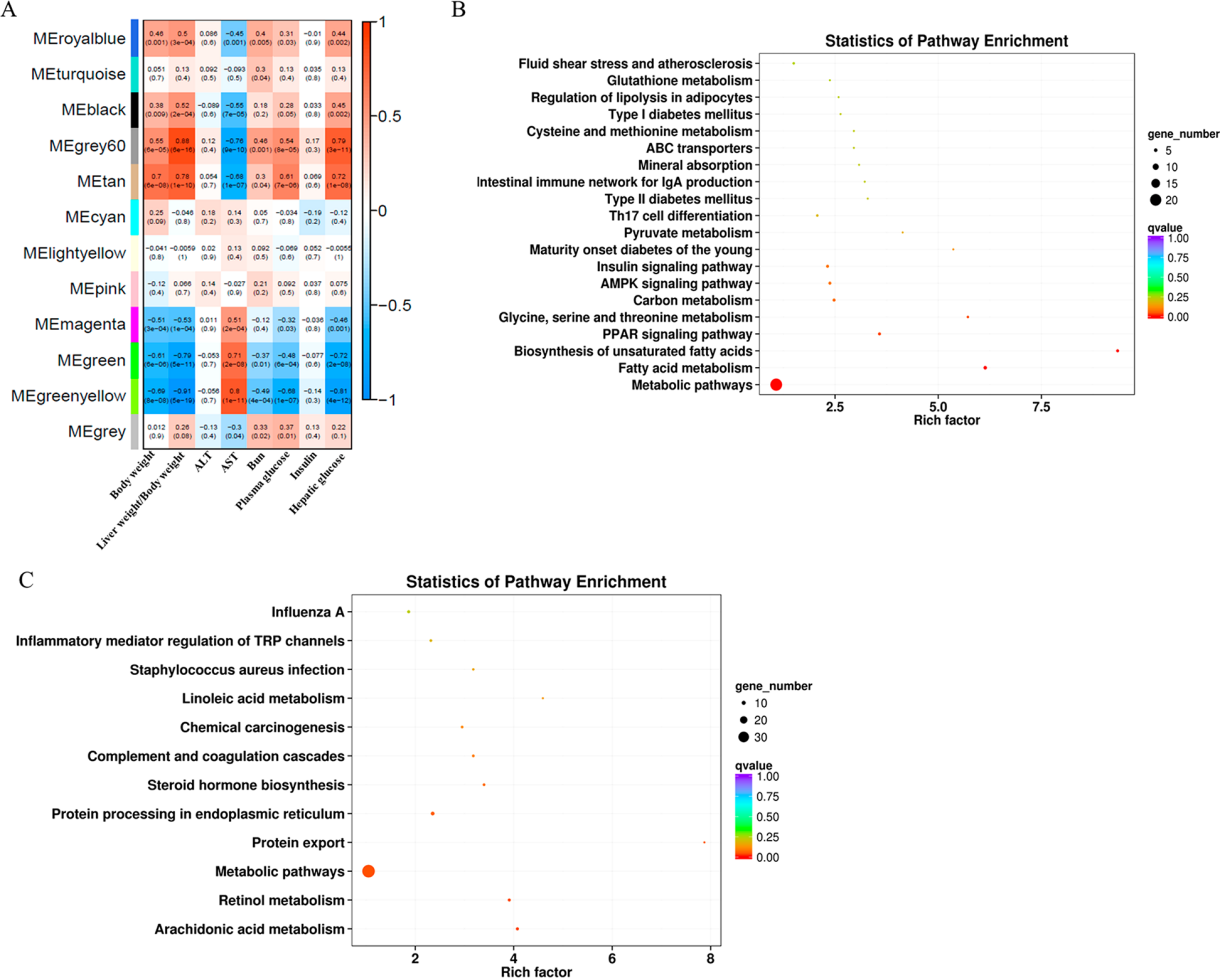
Heatmap of the correlation between clinical traits and module eigengenes (A). Each column corresponds to a clinical trait, and each row corresponds to a module. Each cell contains the correlation coefficients which correspond to the cell color; green represents negative correlation, and red represents positive correlation. The p values are stated in the brackets. KEGG pathway enrichment analysis based on the genes in MEgrey60 and MEtan modules (B). KEGG pathway enrichment analysis based on the genes in MEgreen and MEgreenyellow modules (C).

WGCNA analysis was performed to identify coexpression modules using DEGs and their correlation with clinical traits. Twelve coexpression modules were identified and correlated to various clinical traits reported previously (body weight, AST, ALT, blood urea nitrogen, plasma glucose, and insulin)^8^ and investigated in this study (hepatic glucose and liver/body weight ratio) (Figure 7A). MEgrey60 and MEtan modules showed positive correlation with body weight (Pearson r = 0.55, p = 6 × 10^–5^; r = 0.7, p = 6 × 10^–8^), liver weight/body weight ratio (r = 0.88, p = 6 × 10^–10^; r = 0.78, p = 1 × 10^–10^), plasma glucose (r = 0.54, p = 8 × 10^–5^; r = 0.61, p = 7 × 10^–6^), and hepatic glucose (r = 0.79, p = 3 × 10^–11^; r = 0.72, p = 1 × 10^–8^) and negative correlation with AST (r = −0.76, p = 9 × 10^–10^; r = −0.68, p = 1 × 10^–7^). MEgreen and MEgreenyellow modules showed negative correlation with body weight (r = −0.61, p = 6 × 10^–6^; r = −0.69, p = 8 × 10^–8^), liver weight/body weight ratio (r = −0.79, p = 5 × 10^–11^; r = −0.91, p = 5 × 10^–19^), plasma glucose (r = −0.48, p = 6 × 10^–4^; r = −0.68, p = 1 × 10^–7^), and hepatic glucose (r = −0.72, p = 2 × 10^–8^; r = −0.81, p = 4 × 10^–12^) and positive correlation with AST (r = 0.71, p = 2 × 10^–8^; r = 0.8, p = 1 × 10^–11^). Since genes in MEgrey60 and MEtan modules showed similar correlation to certain clinical traits mentioned above, so did genes in MEgreen and MEgreenyellow modules. Next, genes in MEgrey60 and MEtan modules as well as genes in MEgreen and MEgreenyellow modules were subjected to KEGG pathway enrichment analysis. We found genes in MEgrey60 and MEtan modules were significantly enriched in 20 KEGG pathways (Figure 7B), which were mainly involved in lipid metabolism (fatty acid metabolism, biosynthesis of unsaturated fatty acids, regulation of lipolysis in adipocytes), amino acid metabolism (glycine, serine and threonine metabolism, cysteine and methionine metabolism, glutathione metabolism), diabetes (maturity onset diabetes of the young, type II diabetes mellitus, type I diabetes mellitus, insulin signaling pathway), metabolic pathways, pyruvate metabolism, AMPK signaling pathway, PPAR signaling pathway, and Th17 cell differentiation. Genes in MEgreen and MEgreenyellow modules were enriched in 12 KEGG pathways (Figure 7C) which were mainly involved in arachidonic acid metabolism, retinol metabolism, metabolic pathways, protein export, protein processing in the endoplasmic reticulum, steroid hormone biosynthesis, complement and coagulation cascades, linoleic acid metabolism, and inflammatory mediator regulation of TRP channels.

## Discussion

Liver plays an important role in glucose and lipid metabolism in T2D. A recent review summarized that anthocyanins may have beneficial effects on the diabetic liver by increasing AMP-activated protein kinase phosphorylation (AMPK), protein kinase C phosphorylation, glutathione synthesis, insulin sensitivity, peroxisome proliferator-activated receptor α palmitoyltransferase-1 A, glycogen synthesis, glucose transporter 1 and 4, PI3K/AKT signaling and decreasing, lipogenesis, oxidative damage, acetyl-CoA, gluconeogenesis, and mTOR signaling.^24^ Evidence has shown acylation of anthocyanins contributed differences in stability, antioxidant activity, and bioavailability between nonacylated and acylated anthocyanins; for example, acylated anthocyanins have been reported to have higher inhibitory activity on α-glucosidase than corresponding nonacylated anthocyanins;^25^ acylated anthocyanins showed lower recovery in both urine and plasma compared to their nonacylated counterpart in a bioavailability study in humans, which indicates more acylated anthocyanins could be fermented by gut microbiota.^26^

A recent human postprandial study reported the phenolic compounds in plasma and urine after a meal supplemented with anthocyanins extracted from purple potato variety “Synkeä Sakari”, the same variety as the potato used for the anthocyanin extract in this study,^27^ the low molecular weight phenolic acids and their conjugates were found to be the major phenolic compounds detected in plasma and urine, confirming low absorption efficiency of the acylated anthocyanins in the small intestine and extensive metabolism by gut microbiota.^27^ The metabolites of anthocyanins produced by the action of gut microbiota are known to be more efficiently absorbed than their parent compounds in the gut, likely playing an important role in the health-promoting effects of acylated anthocyanins.

A transcriptomic study showed that a nonacylated anthocyanin pelargonidin-3-*O*-glucoside extracted from wild raspberry had a beneficial effect on the hepatic transcriptome in *db/db* mice through regulating genes involved in glucose and lipid metabolism.^12^

An extract rich in acylated anthocyanins from purple sweet potato increased the acetyl-coenzyme A carboxylase (ACC) and phosphorylation of AMPK in fatty liver.^28^ However, the effect of acylated anthocyanin on the hepatic transcriptome and metabolic profile of the healthy or type 2 diabetic state has not been reported before.

This is the first study to compare the effects of nonacylated anthocyanins from berries and nonacylated anthocyanins from potatoes on the hepatic metabolic profile and transcriptome in diabetes.

High levels of liver weight and liver weight/body weight ratio may suggest the development of the fatty liver. H-AAPP decreased these levels indicating a possible improvement in the state of fatty liver. Among the anthocyanin treatment groups, the H-AAPP group showed the greatest number of DEGs and DETs compared to the M group and fell into the same cluster with the Con and ND groups at the transcript level. Thus, H-AAPP might exert the most beneficial effect on ZDF rats.

Venn diagrams showed the specific genes affected by NAAB and AAPP. *Gstm6*, associated with the glutathione metabolism, drug metabolism, and metabolism of xenobiotics by cytochrome P450, was reported down-regulated in both insulin-resistant and diabetic mice and might be involved in the progression of T2D.^29,30^ Expression of *Gstm6* was restored by both L-NAAB and H-NAAB.

One common gene decreased in the M group compared to the ND and Con groups that was upregulated by both types of anthocyanin extracts (NAAB and AAPP) was *Mgat4a* encoding the N-acetylglucosaminyl transferase GnT-4a. A study has shown that genetic inactivation of the *Mgat4a* gene in mice led to the improper assembling of glucose transporter 2 N-glycan, which caused a failed interaction between glucose transporter 2 and the plasma membrane lectin galectin 9, causing glucose transporter 2 internalization thereby suppressing glucose uptake and glucose-stimulated insulin secretion.^31^ A high-fat diet was also found to inhibit the expression of the *Mgat4a*.^32^ Both the nonacylated anthocyanins from bilberries NAAB and acylated anthocyanins from purple potatoes (AAPP) attenuated the down-regulation of Mgat4a in ZDF rats, indicating the potential role of anthocyanins in restoring the function of glucose transporter 2 in the diabetic state.

Overexpression of *Lpl* encoding lipoprotein lipase in the liver of mice led to a 2-fold increase in hepatic triglyceride content and caused insulin resistance partly due to the impaired response to insulin to suppress endogenous glucose production.^33^ In this study, a high level of *Lpl* in the ZDF rats was downregulated by both L-NAAB and H-NAAB, which may lead to improved insulin sensitivity. Early growth response gene-1 (*Egr1*) can be transiently activated by glucagon in hepatocytes, mediating glucagon-regulated gluconeogenesis.^34^ AAPP decreased *Egr1*, which might have been linked with decreased gluconeogenesis.

In the Con/M comparison, *Fasn, Acly, Acaca*, and *Srebf1* were found as increased hub genes. *Fasn, Acly*, and *Acaca* are target genes of sterol regulatory element-binding protein 1(SREBP-1) encoded by *Srebf1*. Expressions of these genes are required for fatty acid synthesis, which was increased in the M group.^35^ We found three increased hub genes in the M group compared to Con and ND groups, *Gapdh*, *Src*, and *Esr1*. The activity of glyceraldehyde 3-phosphate dehydrogenase (Gapdh) has been shown to be significantly perturbed in diabetes; however, *Gapdh* gene expression can be regulated by hormonal, nutritional, and metabolic factors and was reported increased in the diabetic liver.^36^*Src*, as an essential coordinator of hepatic glucose production, could be activated in type 2 diabetes by G-protein coupled receptors (GCPRs), TGF-beta, and reactive oxygen species (ROS).^37^ Thus, ZDF rats might be characterized by active fatty acid synthesis and low glucose production.

*Fos* and *Jun* are two subunits of activator protein 1 (AP-1) which is a transcriptional regulator. The activity of AP-1 can be regulated by various stimuli, including stress signals, cytokines, infections, and growth factors.^38^ AP-1 was reported to be increased in the ZDF rats because oxidative stress could activate Jun N-terminal kinase (JNK) and further positively regulate AP-1,^39^ and suppression of JNK in diabetic mice has been reported to improve glucose tolerance and insulin resistance.^40^ In our study, *Jun* was increased in the M group compared to the ND group (*P* < 0.05, fold change <2), and the AAPP extracts significantly downregulated *Jun* and *Fos* expression. The high dose bilberry anthocyanin extract (H-NAAB) also downregulated *Jun* expression. Compared to the NAAB extract, the AAPP extract might have better ability for regulating AP-1, alleviating insulin resistance and improving glucose tolerance.

To aid in investigating how these anthocyanin extracts affected the liver of diabetic ZDF rats at the metabolite level, ^1^H NMR metabolomics was performed. Hepatic glucose, lactate, alanine, and pyruvate, which are metabolites in glycolysis, were found to be increased in the M group. These findings are in agreement with the results from the plasma in our previous study.^8^ This might reflect an excess of adaptive glycolysis in type 2 diabetes.^41^ Moreover, lactate, glycerol, alanine, and glutamine are the main gluconeogenic precursors accounting for over 90% of the overall gluconeogenesis.^42^ Altogether, the results indicate an increased level of glycolysis and gluconeogenesis in the M group compared to the control groups of lean rats (Con and ND). All the anthocyanin treatment groups showed decreased levels of glucose, lactate, alanine, pyruvate, and glycerol, and the treatment with acylated anthocyanins (AAPP) additionally decreased the glutamine level, indicating decreased hepatic glucose production and improved glycolysis and gluconeogenesis metabolism in ZDF rats by anthocyanin extracts.

Glucokinase encoded by *Gck* and hepatic pyruvate kinase encoded by *Pklr* are key enzymes that catalyze irreversible steps in glycolysis. The glucokinase level in type 2 diabetes is considered either decreased^43^ or dependent on the insulin and glucagon levels.^44^ In addition to catalyzing the first step in glycolysis, glucokinase is also essential for glycogen synthesis, and glucokinase activators are identified as antidiabetic medicines.^45^

Similar to our results, previous research showed that *Gck* expression was higher in ZDF rats at age 10–11 weeks compared to their lean counterparts, and there was no difference in the glucokinase protein level between ZDF rats and lean Zucker rats.^44^ However, as diabetes progressed in the ZDF rats (from 14 to 28 week of age), both *Gck* and glucokinase protein levels were decreased.^44^ NAAB increased *Gck* expression indicating its potential role as a glucokinase activator. AAPP significantly decreased the expression of *Pklr*, indicating AAPP might decrease glycolysis in the diabetic rat.^46^ Two rate-limiting enzymes in hepatic gluconeogenesis are encoded by *G6pc* (encoding glucose 6-phosphatase catalytic subunit) and *Pck1* (encoding phosphoenolpyruvate carboxykinase). L-AAPP, H-AAPP, and H-NAAB significantly decreased the expression of *G6pc* in the liver, which was consistent with the result obtained by qPCR in our previous study,^8^ and H-AAPP significantly decreased *Pck1*. H-NAAB and AAPP modulated glycolysis and gluconeogenesis in diabetes at the gene expression level.

Evidence has shown that excess branched-chain amino acids (BCAAs) could cause insulin resistance and dysregulation of glucose metabolism.^47^ AAAs, particularly phenylalanine and tyrosine, have been reported to be elevated together with BCAAs in the plasma of patients with obesity-related insulin resistance.^48^ In our study, BCAAs and AAAs in the M group showed an overall increase compared to the Con and ND groups. Upregulation of hepatic BCAAs, in particular, valine and leucine, quantified by amino acid analyzer, were also observed in ZDF rats compared to lean Zucker rats,^49^ which was in agreement with our findings. Moreover, the first step to break down BCAA by branched-chain aminotransferases is considered to occur in extrahepatic organs;^47^ therefore, both the hepatic and plasma levels of BCAAs showed similar changes and elevated in the model group of ZDF rats.^8^ Anthocyanin treatment groups showed decreased levels of hepatic BCAAs, indicating potential improvement in glucose regulation.

The *Bhmt* gene encodes betaine-homocysteine S-methyltransferase (BHMT) which transfers betaine to dimethylglycine. The expression of *Bhmt* was increased in the M group, as was shown in a previous study in ZDF rats,^50^ leading to the increase of hepatic dimethylglycine. In all the treatment groups the elevated levels of dimethylglycine were significantly reversed to the normal state. Decreased expression of *Bhmt* was also observed in all the anthocyanin treatment groups, especially in the AAPP groups showing a statistically significant decrease.

A recent review summarized that excessive substrates of metabolic energy such as carbohydrates and fatty acids exceeding the hepatic capacity to process are the pathogenic driver in the development of nonalcohol fatty liver and insulin resistance.^51^ In this study, the M groups showed an overall increased lipid profile such as high levels of phospholipid, triglyceride, fatty acid, and unsaturated fatty acids, all contributing to the development of fatty liver. The L-AAPP and H-AAPP groups slightly improved the hepatic lipid profile by regulating fatty acid residues and unsaturated fatty acids. We examined genes in the biosynthesis of the unsaturated fatty acid pathway from the KEGG pathway library, and an overall upregulation of the genes in this pathway was shown in the M group. The high dose potato anthocyanin (H-AAPP) group showed decreased *Acox1* expression. Previously, it has been shown that a high-fat diet may increase the expression of *Acox* gene encoding peroxisomal acyl-coenzyme A oxidase, which catalyzes the first step of peroxisomal beta-oxidation.^52^ Inhibition of *Acox1* could increase hepatic mitochondrial fatty acid oxidation and decrease hepatic lipid and ROS contents; therefore, downregulation or inhibition of *Acox1* expression is an effective approach for the treatment of HFD or obesity-induced metabolic diseases.^52^ Additionally, *Acox1*^*–/–*^*/ob/ob* mice were found resistant to obesity.^53^ A high dose of AAPP might exert a beneficial effect through inhibiting *Acox1*.

In conclusion, our study provides the first multiomics analysis of the effect of acylated anthocyanins from purple potato and nonacylated anthocyanins from bilberry on the hepatic metabolism in ZDF rats. Among all anthocyanin treatment groups, the group treated with high dose potato anthocyanins (H-AAPP) showed transcriptomic and metabolic profiles closer toward the lean Zucker rats. We found both anthocyanin extracts, especially AAPP, had beneficial effects by restoring metabolites and expression of genes involved in glycolysis, decreasing AP-1 at the gene expression level. Besides acylation being the one factor that affects the bioavailability and biological activities of acylated anthocyanins and nonacylated anthocyanins, the varieties of anthocyanin metabolites determined by the type of aglycone of anthocyanins and acyl group as well as the composition of gut microbiota might play an even more important role. Overall, our study showed that acylated anthocyanins extracted from purple potato which is an affordable anthocyanin source have a better modulatory effect on hepatic transcriptomic and metabolic profiles in T2D compared to nonacylated anthocyanins from bilberry.

## Supporting Information Available

KEGG annotation of genes that are affected by anthocyanin extracts, bins for quantification of metabolites, ^1^H chemical shift assignments of the metabolites, fold change of the metabolites compared to the M group, functionally grouped networks of enriched GO terms, 2D ^1^H NMR spectra for hepatic aqueous and lipid extracts, and several hepatic genes expression. (PDF)

## Abbreviations Used

AAAs: aromatic amino acids
AAPP: acylated anthocyanins extract from purple potatoes
BCAAs: branched-chain amino acids
NAAB: nonacylated anthocyanins extract from bilberries
ZDF rat: Zucker diabetic fatty rat
WGCNA: weighted gene coexpression network analysis
KEGG: Kyoto Encyclopedia of Genes and Genomes
GO: gene ontology
