# Supplementary material for: 1H NMR Metabolomics and Full-Length RNA-Seq Reveal Effects of Acylated and Nonacylated Anthocyanins on Hepatic Metabolites and Gene Expression in Zucker Diabetic Fatty Rats
Source: J Agric Food Chem. 2021 Apr 9;69(15):4423–37. doi: 10.1021/acs.jafc.1c00130 (PMC8154569; doi:10.1021/acs.jafc.1c00130)
Supplement: Supplementary file 1 — jf1c00130_si_001.pdf [file jf1c00130_si_001.pdf]

^{1}H NMR Metabolomics and Full-Length RNA-Seq Reveal Effects of Acylated and Nonacylated Anthocyanins on Hepatic Metabolites and Gene Expression in Zucker Diabetic Fatty Rats

Kang Chen[†], Xuetao Wei[‡], Raghunath Pariyani[†], Maaria Kortesniemi[†], Yumei Zhang[§*], Baoru Yang[†*]

[†]Food Chemistry and Food Development, Department of Life Technologies, University of Turku, FI-20014 Turun yliopisto, Finland

[‡]Beijing Key Laboratory of Toxicological Research and Risk Assessment for Food Safety, Department of Toxicology, School of Public Health, Beijing University, Beijing 100191, China

[§]Department of Nutrition and Food Hygiene, School of Public Health, Beijing University, Beijing 100191, China

Table S1. Genes restored by anthocyanin extracts in ZDF rats and their annotation and involved KEGG pathways.

Gene ID	Gene type	Gene symbol	KEGG_annotation
ENSRNOG00000016593	Known_gene	Pde6c	K13757 cone cGMP-specific 3',5'-cyclic phosphodiesterase subunit alpha
ENSRNOG00000003657	Known_gene	Pkmyt1	K06633 membrane-associated tyrosine- and threonine-specific cdc2-inhibitory kinase
ENSRNOG00000012181	Known_gene	Lpl	K01059 lipoprotein lipase
ENSRNOG00000019661	Known_gene	Gdf15	K05504 growth differentiation factor 15
ENSRNOG00000000257	Known_gene	Smpd3	K12352 sphingomyelin phosphodiesterase 3
ENSRNOG00000011402	Known_gene	Isg20l2	K18329 interferon-stimulated 20 kDa exonuclease-like 2
ENSRNOG00000019017	Known_gene	Gstm6	K00799 glutathione S-transferase
ENSRNOG00000028749	Known_gene	Atxn7l2	K11318 ataxin-7
ENSRNOG00000008360	Known_gene	LOC103692167	K11487 polycomb group RING finger protein 1
ENSRNOG00000011156	Known_gene	Clstn3	--
ENSRNOG00000011329	Known_gene	Pkm	K00873 pyruvate kinase, muscle
ENSRNOG00000015297	Known_gene	RGD1561662	--
ENSRNOG00000018276	Known_gene	Mgat4a	K00738 alpha-1,3-mannosylglycoprotein beta-1,4-N-acetylglucosaminyltransferase A/B
ENSRNOG00000002419	Known_gene	Plp1	K17271 myelin proteolipid protein
ENSRNOG00000019422	Known_gene	Egr1	K09203 early growth response protein 1
ONT.2489	New_gene	ONT.2489	--
ONT.9104	New_gene	ONT.9104	--
ONT.13355	New_gene	ONT.13355	--
ONT.5923	New_gene	ONT.5923	--
ONT.6346	New_gene	ONT.6346	--
ONT.7547	New_gene	ONT.7547	--
ONT.3213	New_gene	ONT.3213	K03131 transcription initiation factor TFIID subunit 6
ONT.1624	New_gene	ONT.1624	--
ONT.6633	New_gene	ONT.6633	--
ONT.4092	New_gene	ONT.4092	--
ONT.5347	Novel_lncRNA_gene	ONT.5347	--

Table S2. Hub genes identified in the six comparisons: Con/M, ND/M, L-NAAB/M, H-NAAB/M, L-AAPP/M, H-AAPP/M. The degree of connectivity was calculated by protein-protein interaction based on STRING.

Gene ID	Gene_type	Gene symbol	Regulated	Degree	KEGG_annotation
**Con vs M**					
ENSRNOG00000018630	Known_gene	Gapdh	Down	39	K00134 glyceraldehyde 3-phosphate dehydrogenase
ENSRNOG00000009495	Known_gene	Src	Down	39	K05704 tyrosine-protein kinase Src
ENSRNOG00000045636	Known_gene	Fasn	Down	30	K00665 fatty acid synthase, animal type
ENSRNOG00000019322	Known_gene	Notch1	Up	29	K02599 Notch 1
ENSRNOG00000003463	Known_gene	Srebf1	Down	27	K07197 sterol regulatory element-binding transcription factor 1
ENSRNOG00000016924	Known_gene	Acly	Down	27	K01648 ATP citrate (pro-S)-lyase
ENSRNOG00000019358	Known_gene	Esr1	Down	25	K08550 estrogen receptor alpha
ENSRNOG00000007350	Known_gene	Rac2	Up	23	K07860 Ras-related C3 botulinum toxin substrate 2
ENSRNOG00000034013	Known_gene	Acaca	Down	23	K11262 acetyl-CoA carboxylase / biotin carboxylase 1
ENSRNOG00000047945	Known_gene	Cyp2c13	Down	21	K07413 cytochrome P450 family 2 subfamily C
**ND vs M**					
ENSRNOG00000014288	Known_gene	Fn1	Up	58	K05717 fibronectin 1
ENSRNOG00000018630	Known_gene	Gapdh	Down	52	K00134 glyceraldehyde 3-phosphate dehydrogenase
ENSRNOG00000019974	Known_gene	Uba52	Up	50	K02927 large subunit ribosomal protein L40e
ENSRNOG00000009495	Known_gene	Src	Down	50	K05704 tyrosine-protein kinase Src
ENSRNOG00000018294	Known_gene	Hspa5	Up	47	K09490 heat shock 70kDa protein 5
ENSRNOG00000015079	Known_gene	Ddost	Up	41	K12670 oligosaccharyltransferase complex subunit beta
ENSRNOG00000018445	Known_gene	Agt	Up	39	K09821 angiotensinogen
ENSRNOG00000024848	Known_gene	Fga	Up	38	K03903 fibrinogen alpha chain
ENSRNOG00000013741	Known_gene	Ube2d3	Up	38	K06689 ubiquitin-conjugating enzyme E2 D
ENSRNOG00000003343	Known_gene	Esr1	Up	37	K08550 estrogen receptor alpha
**L-NAAB vs M**					
ENSRNOG00000011329	Known_gene	Pkm	Down	4	K00873 pyruvate kinase
ENSRNOG00000009565	Known_gene	Pdk4	Down	4	K00898 pyruvate dehydrogenase kinase 2/3/4
ENSRNOG00000059593	Known_gene	Cetn2	Down	3	K10840 centrin-2
ENSRNOG00000003600	Known_gene	Pnpt1	Down	3	K00962 polyribonucleotide nucleotidyltransferase
ENSRNOG00000010805	Known_gene	Fabp4	Down	3	K08753 fatty acid-binding protein 4, adipocyte
ENSRNOG00000018276	Known_gene	Mgat4a	Up	3	K00738 alpha-1,3-mannosylglycoprotein beta-1,4-N-acetylglucosaminyltransferase A/B
ENSRNOG00000021264	Known_gene	C5ar1	Down	2	K04010 C5a anaphylatoxin chemotactic receptor
ENSRNOG00000007890	Known_gene	Nubpl	Down	2	K03593 ATP-binding protein involved in chromosome partitioning
ENSRNOG00000021157	Known_gene	Ogfrl1	Down	2	-
ENSRNOG00000016219	Known_gene	Arhgap9	Down	2	K20634 Rho GTPase-activating protein 9
**H-NAAB vs M**					
ENSRNOG00000026293	Known_gene	Jun	Down	8	K04448 transcription factor AP-1
ENSRNOG00000019358	Known_gene	Esr1	Down	7	K08550 estrogen receptor alpha
ENSRNOG00000019422	Known_gene	Egr1	Down	6	K09203 early growth response protein 1

ENSRNOG00000014117	Known_gene	Hmox1	Down	6	K00510 heme oxygenase (biliverdin-producing)
ENSRNOG00000003745	Known_gene	Atf3	Down	5	K09032 activating transcription factor 3
ENSRNOG00000030644	Known_gene	Mt-nd1	Down	5	K03878 NADH-ubiquinone oxidoreductase chain 1
ENSRNOG00000042838	Known_gene	Junb	Down	5	K09028 transcription factor jun-B
ENSRNOG00000012181	Known_gene	Lpl	Down	5	K01059 lipoprotein lipase
ENSRNOG00000020990	Known_gene	Fgf21	Down	5	K04358 fibroblast growth factor
ENSRNOG00000031979	Known_gene	Mt-atp6	Down	4	K02126 F-type H+-transporting ATPase subunit a
**L-AAPP vs M**					
ENSRNOG00000008015	Known_gene	Fos	Down	7	K04379 proto-oncogene protein c-fos
ENSRNOG00000019422	Known_gene	Egr1	Down	6	K09203 early growth response protein 1
ENSRNOG00000007159	Known_gene	Ccl2	Up	5	K14624 C-C motif chemokine 2
ENSRNOG00000003977	Known_gene	Dusp1	Down	5	K21278 dual specificity protein phosphatase 1
ENSRNOG00000003300	Known_gene	Btg2	Down	5	K14443 protein Tob/BTG
ENSRNOG00000026293	Known_gene	Jun	Down	5	K04448 transcription factor AP-1
ENSRNOG00000009565	Known_gene	Pdk4	Down	4	K00898 pyruvate dehydrogenase kinase 2/3/4
ENSRNOG00000011329	Known_gene	Pkm	Down	3	K00873 pyruvate kinase
ENSRNOG00000013000	Known_gene	Ldhb	Down	3	K00016 L-lactate dehydrogenase
ENSRNOG00000007323	Known_gene	Opn3	Down	2	K04256 c-opsin
**H-AAPP vs M**					
ENSRNOG00000007159	Known_gene	Ccl2	Up	15	K14624 C-C motif chemokine 2
ENSRNOG00000008015	Known_gene	Fos	Down	13	K04379 proto-oncogene protein c-fos
ENSRNOG00000026293	Known_gene	Jun	Down	13	K04448 transcription factor AP-1
ENSRNOG00000016571	Known_gene	Ngf	Down	12	K02582 nerve growth factor, beta
ENSRNOG00000015036	Known_gene	Ctgf	Up	9	K06827 connective tissue growth factor
ENSRNOG00000000853	Known_gene	Aif1	Down	9	K18617 allograft inflammatory factor 1
ENSRNOG00000003622	Known_gene	Cybb	Down	8	K08008 NADPH oxidase
ENSRNOG00000019422	Known_gene	Egr1	Down	8	K09203 early growth response protein 1
ENSRNOG00000012807	Known_gene	C1qa	Down	7	K03986 complement C1q subcomponent subunit A
ENSRNOG00000013973	Known_gene	Anxa2	Up	6	K17092 annexin A2

Table S3. Chemical shift assignments of the metabolites observed in the ^{1}H NMR spectra of aqueous extract from rat liver using one dimensional (1D) NOESY, two-dimensional HSQC, *J*-resolved spectra.

No.	Metabolites	Assignments	δ1H (multiplicity)	δ13C	J value (Hz)	Methods	Bins used for quantification
1	Cholate	αCH	0.71(s)	-	-	NOESY ,HSQC	0.70-0.72
2	Isoleucine	αCH,	0.94(t),	19.9	6.9	NOESY, HSQC	0.99-1.01
3	Valine	αCH, βCH,	0.98(d).1.03(d),	19.4	7.01,7.05	NOESY,HSQC,JRES	1.02-1.04
4	Leucine	αCH,	0.92(d),)	19.9	6.72	NOESY, HSQC	0.935-0.965
5	3-Hydroxybutyrate	αCH,	1.18(d)	24.9	6.31	NOESY.HSQC,JRES	1.18-1.20
6	Lactate	αCH, βCH3	1.32(d), 4.11(q)	22.97(d),74.42(m)	7.00	NOESY, HSQC,JRES	1.30-1.34
7	Alanine	βCH3	1.47(d)	18.9	7.14	NOESY, HSQC,JRES	1.45-1.48
8	Lysine	γCH3	1.89(m)	32.5	15.2	NOESY, HSQC,JRES	3.00-3.02
9	Acetate	βCH3	1.91(s)	26.08	-	NOESY,HSQC, JRES	1.904-1.914
10	Glutamine	γCH2	2.44(m)	33.50	-	NOESY, JRES	2.445-2.455
11	Glutamate	βCH2, γCH	2.05(m),2.34(m)	29.80	-	NOESY, JRES	2.02-2.08
12	Methione	βCH2	2.64(t)	31.57	14.85	NOESY,HSQC, JRES	2.125-2.130

13	Glutathione	5-CH2	2.55(m)	34.05	-	NOESY,HSQC	2.56-2.59
14	Pyruvate	CH3	2.36(s)	29.22	-	NOESY,HSQC, JRES	2.362-2.365
15	Succinate	CH2	2.38	36.80	-	NOESY,HSQC, JRES	2.39-2.40
16	Creatine	N-CH3, CH2	3.02(s), 3.93(s)	41.7, 57.1	-	NOESY,HSQC, JRES	3.024-3.028
17	Aspartate	βCH2	2.72(m)	39.40	-	NOESY,HSQC, JRES	2.78-2.80
18	Malate	βCH2	2,65(m)	45.46	-	NOESY,HSQC, JRES	2.67-2.68
19	Dimethylamine	CH3	2.72(s)	-	-	NOESY, JRES	2.705-2.715
20	Dimethylglycine	CH3	2.91(s)	-	-	NOESY, JRES	2.912-2.918
21	Choline	N(CH3)3	3.19(s)	56.7	-	NOESY,HSQC,JRES	3.18-3.20
22	Phosphocholine	N(CH3)3	3.20(s)	56.52		NOESY,HSQC, JRES	3.204-3.213
23	Taurine	βCH2	3.24	-	14.13	NOESY, JRES	3.259-3.264
24	Maltose	17-CH	3.26	76.70	7.94	NOESY,HSQC, JRES	3.265-3.290
25	Glycine	αCH2	3.54(s)	44.30	-	NOESY,JRES	3.548-3.554
26	Glycerol	CH2	3.55	65.40	-	NOESY,HSQC, JRES	3.630-3.645
27	Mannose	αCH, βCH	5.17(d)	96.8	2.78	NOESY,HSQC	5.170-5.183
28	Glucose	βCH	5.22(d)	94.93	4.82	NOESY,HSQC,JRES	4.62-4.64

							5.21-5.24
29	Uracil	γCH	5.80(d)	-	8.0	NOESY , JRES	5.78-5.79
30	Xanthosine	αCH	7.86(s)	141	-	NOESY,HSQC, JRES	5.830-5.855
31	Uridine	αCH	7.85(d)	144.55	8.15	NOESY,HSQC, JRES	5.895-5.910
32	Fumarate	αCH	6.50(s)	138.00	-	NOESY,HSQC, JRES	6.50-6.52
33	Tyrosine	C2,6H,Ring	6.88(d)	118.89	8.8	NOESY	6.87-6.91
34	Histidine	βCH	7.13(s)	119.99	-	NOESY	7.05-7.08
35	Phenylalanine	C2,6H,Ring;C3,5H,Ring	7.41(m)	130.42,132.11	-	NOESY	7.30-7.44
36	Formate	HCOO-	8.48(s)	-	-	NOESY	8.44-8.45

Table S4. Chemical shift assignments of the metabolites observed in the ^{1}H NMR spectra of lipid extract from rat liver using one dimensional (1D) NOESY, two-dimensional HSQC, *J*-resolved spectra.

No.	Metabolites	Assignments	δ1H (multiplicity)	δ13C	J value (Hz)	Methods	Bins for relative quantification
1	Cholesterol	Chol-$C_{18}H_3$	0.66 (s)	12.00	-	NOESY ,HSQC	-
2	Cholesterol	Chol-$C_{26/27}H_3$	0.84 (dd)	22.00	-	NOESY,HSQC	-
3	FA residue	FA-ω-CH_3	0.88(t),	14.10	6.9	NOESY, HSQC, JRES	-
4	Cholesterol	Chol-$C_{21}H_3$	0.92(d)	19.40	7.01,7.05	NOESY,HSQC	0.92
5	Omega-3 Fatty acid						
	(DHA+ EPA+ linolenic)	ω-3 **CH**3-CH2-C=C	0.96(t)	14.20	7.5	NOESY, HSQC	0.96
6	Cholesterol	Chol-$C_{19}H_3$	1.00(d)	19.90	6.31	NOESY.HSQC,JRES	-
7	FA residue	-(CH2)n-	1.20–1.40(b)	-	-	NOESY, HSQC,JRES	1.22-1.28
8	FA residue	C**H₂**-CH₂-CO	1.60 (m)	24.70	7.14	NOESY, HSQC,JRES	-
9	FA, ARA+EPA	βH2	1.70(m)	24.90	-	NOESY, HSQC,JRES	1.66-1.68
10	FA, Oleic acid	-CH=CH-C**H₂**-	2.00(m)	26.08	-	NOESY,HSQC	2.00
11	FA, ARA+EPA	γCH2	2.10(m)	26.50	-	NOESY,HSQC, JRES	2.08-2.10
12	Monoglycerides	FA RH -C**H**2-CO-O-C2	2.26(t)	34.10	7.5	NOESY, JRES	2.26

13	FA, αH2	αH2 -C**H2**-CO-OR	2.32(m)	33.9	-	NOESY, JRES	-
14	FA, DHA	αH2 and βH2	2.36(m)	-	-	NOESY,HSQC, JRES	2.36
15	FA, Linoleic acid	-CH=CH-**CH2**-(CH=CH-CH2-)n, n=1	2.76(t)	25.00	6.4	NOESY,HSQC	2.76
16	FA, PUFA	-CH=CH-**CH2**-(CH=CH-CH2-)n, N≥2	2.80(m)	25.59	-	NOESY,HSQC, JRES	2.80-2.82
17	Phosphatidylethanolamine	Alkyl-PE (Phosphoether) R-PO-CH-**CH2**-N-(CH3)3	3.16(m)	40.78	-	NOESY,HSQC, JRES	3.14-3.16
18	Sphingomyelin	-CH2-N-(C**H3**)3	3.30(s)	54.80	-	NOESY,HSQC, JRES	-
19	Phosphatidylcholine	-CH2-N-(**CH3**)3	3.34(s)	54.80	-	NOESY,HSQC, JRES	3.32-3.34
20	Phosphatidylcholine	-	3.80(s)	66.66		NOESY, HSQC, JRES	3.78-3.82
21	Triglyceride	-	3.83(m)	62.45	-		-
22	Total phospholipid	**C3H2(s)**	3.98(s)	63.57		NOESY, JRES	3.92-3.98
23	Triglyceride	Glycerol (C1-**H)** and (C3-**H)**	4.22(dd)	62.1	11.8,6.1	NOESY,HSQC, JRES	-

24	Monoglycerides	Glycerol (C2-H) acylated in pos c2	4.92(m)	75.16	-	NOESY,JRES	4.90-4.92
25	1,2-Diglycerides	Glycerol (C2-H)	5.08(a quartet of doublets)	72.08	-	NOESY,HSQC, JRES	5.08
26	Triglyceride	Glycerol (C2-H)	5.26(m)	68.90	5.1	NOESY,JRES	5.24-5.26
27	Sphingomyelin	CH3(CH2)n-Cb**H**=CaH-C1HOH-	5.68(m)	-	6.7	NOESY,HSQC, JRES	5.88
28	Plasmalogen	CH3(CH2)n-CbH=Ca**H**-C1**H**2OR-	5.88(d)	-	-	NOESY,JRES	5.66-5.68
29	Phosphatidylserine	**NH3**+-CH-COO	6.66(m)	133.60	-	NOESY,HSQC	6.60-6.64

FA, Fatty acid; DHA, docosahexaenoic acid; EPA, dcosapentaenoic acid; ARA, arachidonic acid; PUFA, polyunsaturated fatty acids.

Table S5 Fold change of the aqueous metabolites compared to the M group.

	M/L-NAAB	M/H-NAAB	M/L-AAPP	M/H-AAPP	M/Con	M/ND
Glutamine/Glutamate	1.05	0.94	1.02	1.16	2.07**	2.32 ***
Cholate	1.03	1.10	1.20	1.00	1.18	1.57*
Leucine	1.00	1.48***	1.30**	1.23*	1.15	1.25*
Isoleucine	0.99	1.46***	1.29**	1.20	1.18*	1.30**
Valine	1.01	1.50***	1.31**	1.24*	1.13	1.23*
3-Hydoxybutyrate	0.99	1.52	1.13	0.59*	1.14	0.98
Lactate	1.14*	1.40**	1.40**	1.14*	1.78***	2.28***
Alanine	1.26**	1.51**	1.45**	1.19	1.56**	1.58***
Acetate	1.05	1.41***	1.18	1.00	0.95	0.86
Methionine	1.00	1.41**	1.37**	1.40*	1.26	1.35**
Glutamate	1.05	1.41**	1.44**	1.41*	1.23	1.29**
Pyruvate	1.43**	1.41**	1.91***	1.18	1.11	1.50***
Succinate	1.08	1.17	1.51	1.23	1.62	2.06*
Glutamine	1.08	1.32	1.42*	1.59**	2.47***	2.96***
Glutathione	1.09	1.21	1.48*	1.18	1.24	1.45
Malate	1.34	1.69**	1.29	1.18	1.22	1.19
Dimethylamine	1.11	1.40*	1.21	1.03	1.61**	2.35***
Aspartate	1.08	1.56*	1.31	1.26	1.00	0.98
Dimethylglycine	1.50	1.96**	1.73*	1.61*	1.94**	2.02**
Lysine	1.07	1.54***	1.36*	1.30**	1.12	1.02
Creatine	1.01	1.44***	1.31*	1.23	1.25*	1.06
Choline	1.17	1.38	1.53*	1.19	0.65***	0.51***
Phosphocholine	1.16	1.21	1.15	0.96	1.10	1.35*
Taurine	1.26*	1.38	1.49*	1.17	1.89**	1.98***
Maltose	1.09	1.54**	1.47*	1.63***	4.18***	4.97***
Glycine	1.07	1.48***	1.41***	1.38***	1.41***	1.28***
Glycerol	1.08	1.54***	1.49***	1.65**	2.42***	2.27***
Mannose	1.24**	1.58***	1.50***	1.38**	1.71***	2.18***
Glucose	1.16	1.40***	1.49***	1.39**	2.92***	4.48***
Uracil	1.10	1.52*	1.33**	1.30*	1.09	1.05
Xanthosine	0.91	0.96	1.16	1.08	0.97	1.13
Uridine	1.15	1.14	1.21*	0.94	1.00	1.39*
Cytidine	1.21**	1.47**	1.50***	1.18	1.11	1.14
Fumarate	1.56*	2.07*	1.39	1.10	1.28	1.22
Tyrosine	1.06	1.60**	1.33**	1.32**	1.27**	1.28**
Histidine	1.08	1.47***	1.38**	1.23**	1.07	1.15
Phenylalanine	0.93	1.44**	1.22	1.20*	1.11	1.27*

*p<0.05, **p<0.01, ***p<0.001 as compared with M group

Table S6 Fold change of the lipid metabolites compared to the M group.

	M/L-NAAB	M/H-NAAB	M/L-AAPP	M/H-AAPP	M/Con	M/ND
Plasmalogen	0.97	1.04	0.87	1.05	1.05	0.96
Sphingomyelin	1.05	0.93	1.25	1.19	1.39*	1.39*
Triglyceride	1.04	0.95	0.97	0.98	1.88***	2.28***
Diglyceride	1.11	1.14	1.02	1.06	1.11	1.13
Total phospholipids	1.04	1.01	1.00	1.14	1.28**	1.33**
Phosphatidylcholine	1.05	1.00	1.08	1.16	1.48	1.56
Phosphatidylethanolamine	1.00	1.08	0.96	1.17	1.08	1.02
PUFA	1.04	0.94	1.13	1.18*	1.63***	1.59***
Linoleic acid	1.08	1.00	1.20	1.16	1.16	1.19
DHA	1.14	0.99	1.17	1.12	2.64***	2.75***
Monoglyceride	1.07	1.05	1.30**	1.26*	1.55***	1.51***
ARA+EPA	1.05	0.95	1.13*	1.18*	1.40***	1.37**
Oleic acid	0.93	0.92	1.17	0.98	1.51**	1.43*
Fatty acid residue (FA, -(CH2)n-)	1.03	0.93	1.16	1.12	1.46**	1.46**
Omega3 Fatty acid	1.04	0.97	1.13	1.18**	2.04***	2.35***
Cholesterol	0.95	0.97	1.02	1.12	1.09	1.15
Phosphatidylserine	1.25	2.21	0.87	1.59	0.43**	0.37***
PC/PE	1.21	1.02	1.33	1.00	1.63**	1.82***

FA, Fatty acid; DHA, docosahexaenoic acid; EPA, dcosapentaenoic acid; ARA, arachidonic acid; PUFA, polyunsaturated fatty acids. *p<0.05, **p<0.01, ***p<0.001 as compared with M grou

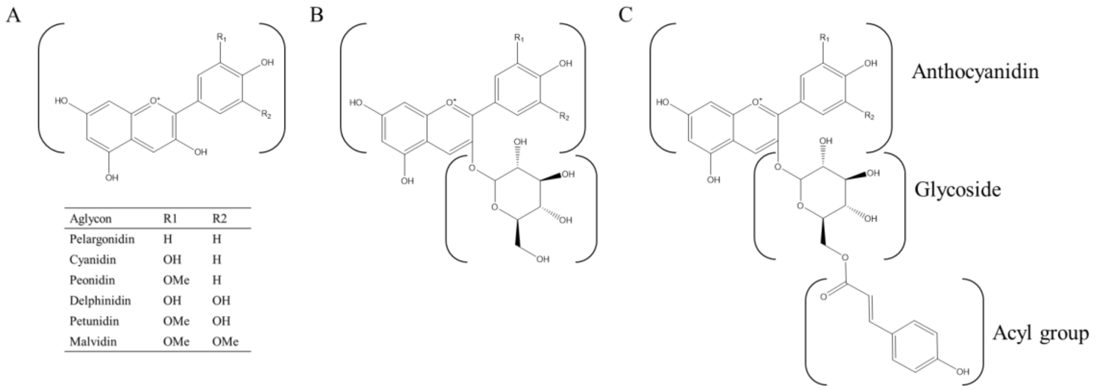

Aglycon	R1	R2
Pelargonidin	H	H
Cyanidin	OH	H
Peonidin	OMe	H
Delphinidin	OH	OH
Petunidin	OMe	OH
Malvidin	OMe	OMe

Figure 1. The skeletal structure of anthocyanidin (A) and examples of nonacylated (B) and acylated (C) anthocyanin.

A Con vs M

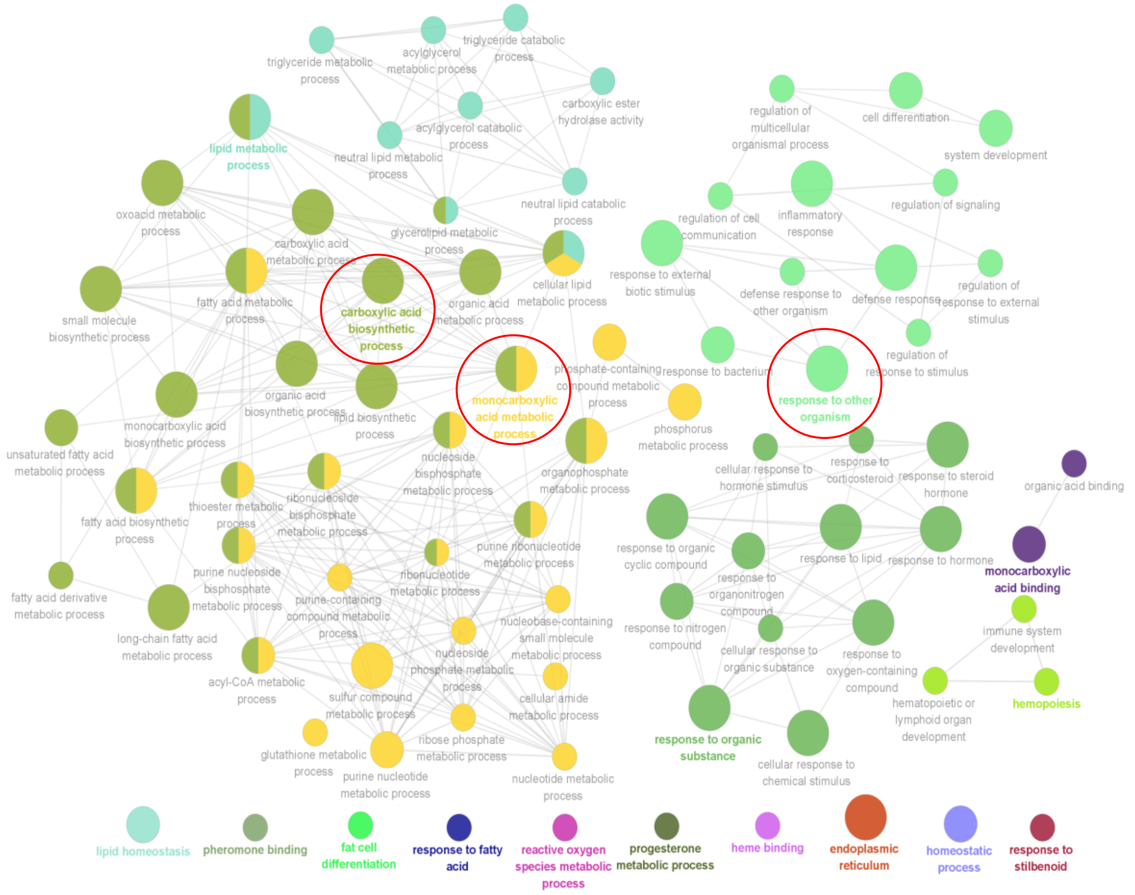

triglyceride metabolic process
acylglycerol metabolic process
triglyceride catabolic process
carboxylic ester hydrolase activity
acylglycerol catabolic process
neutral lipid metabolic process
lipid metabolic process
oxoacid metabolic process
carboxylic acid metabolic process
glycerolipid metabolic process
neutral lipid catabolic process
cellular lipid metabolic process
organic acid metabolic process
small molecule biosynthetic process
fatty acid metabolic process
carboxylic acid biosynthetic process
monocarboxylic acid metabolic process
phosphate-containing compound metabolic process
phosphorus metabolic process
organic acid biosynthetic process
lipid biosynthetic process
monocarboxylic acid biosynthetic process
unsaturated fatty acid metabolic process
fatty acid biosynthetic process
thioester metabolic process
ribonucleoside bisphosphate metabolic process
nucleoside bisphosphate metabolic process
organophosphate metabolic process
purine ribonucleotide metabolic process
ribonucleotide metabolic process
purine nucleoside bisphosphate metabolic process
purine-containing compound metabolic process
fatty acid derivative metabolic process
long-chain fatty acid metabolic process
acyl-CoA metabolic process
nucleobase-containing small molecule metabolic process
nucleoside phosphate metabolic process
sulfur compound metabolic process
cellular amide metabolic process
glutathione metabolic process
ribose phosphate metabolic process
purine nucleotide metabolic process
nucleotide metabolic process
regulation of multicellular organismal process
cell differentiation
system development
regulation of cell communication
inflammatory response
regulation of signaling
response to external biotic stimulus
defense response to other organism
defense response
regulation of response to external stimulus
response to bacterium
response to other organism
regulation of response to stimulus
cellular response to hormone stimulus
response to corticosteroid
response to steroid hormone
organic acid binding
response to organic cyclic compound
response to organonitrogen compound
response to lipid
response to hormone
monocarboxylic acid binding
response to nitrogen compound
cellular response to organic substance
response to oxygen-containing compound
immune system development
hematopoietic or lymphoid organ development
hemopoiesis
response to organic substance
cellular response to chemical stimulus
lipid homeostasis
pheromone binding
fat cell differentiation
response to fatty acid
reactive oxygen species metabolic process
progesterone metabolic process
heme binding
endoplasmic reticulum
homeostatic process
response to stilbenoid

B   ND vs M

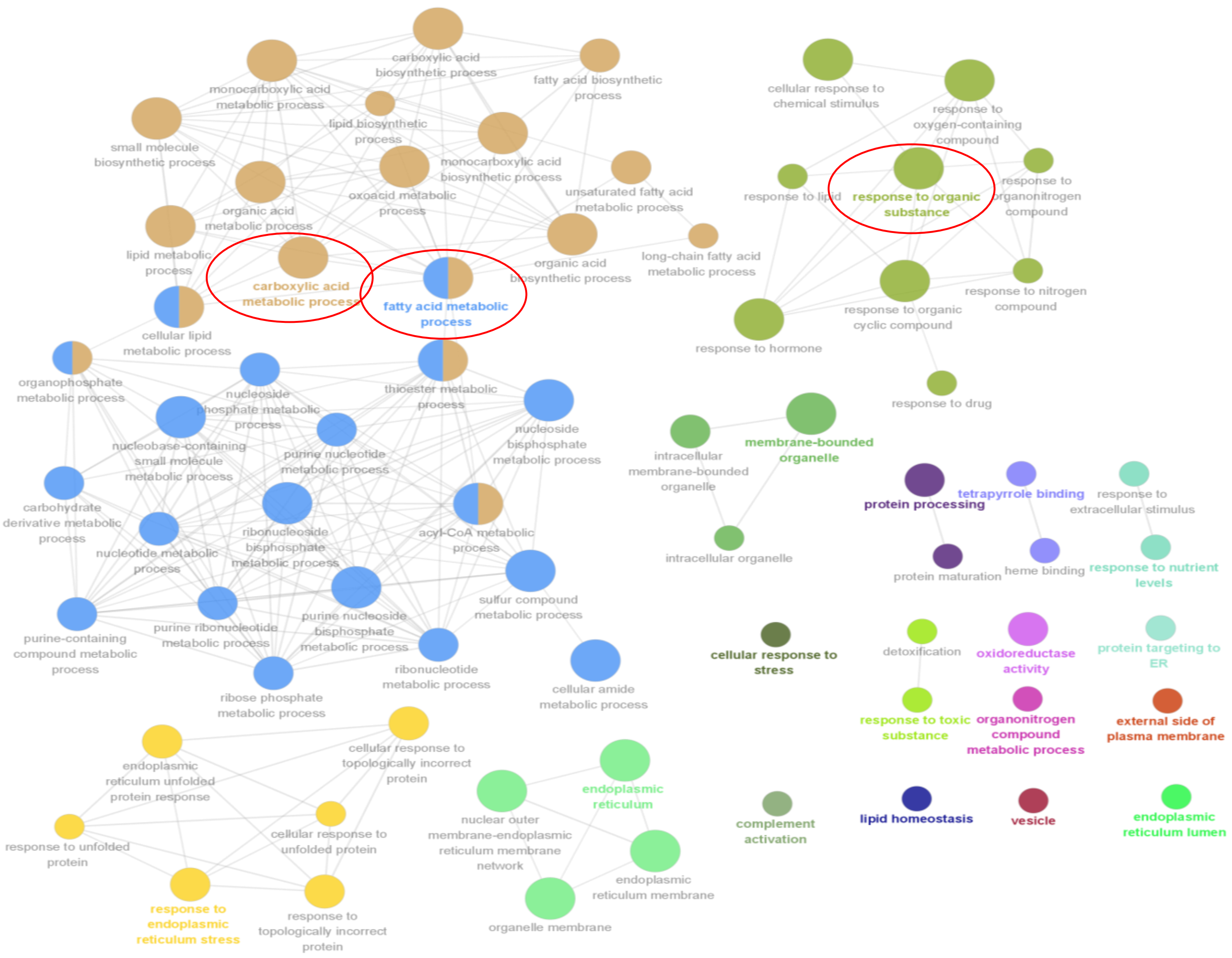

C   L-NAAB vs M

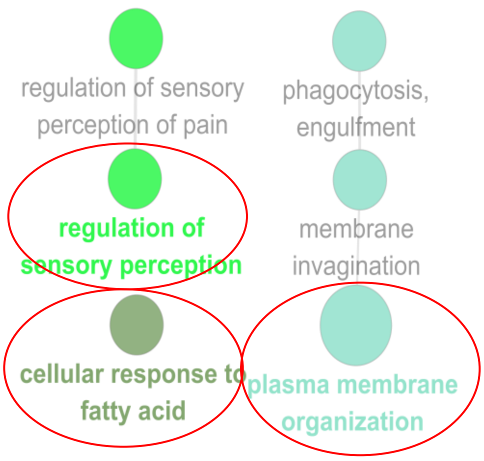

D H-NAAB vs M

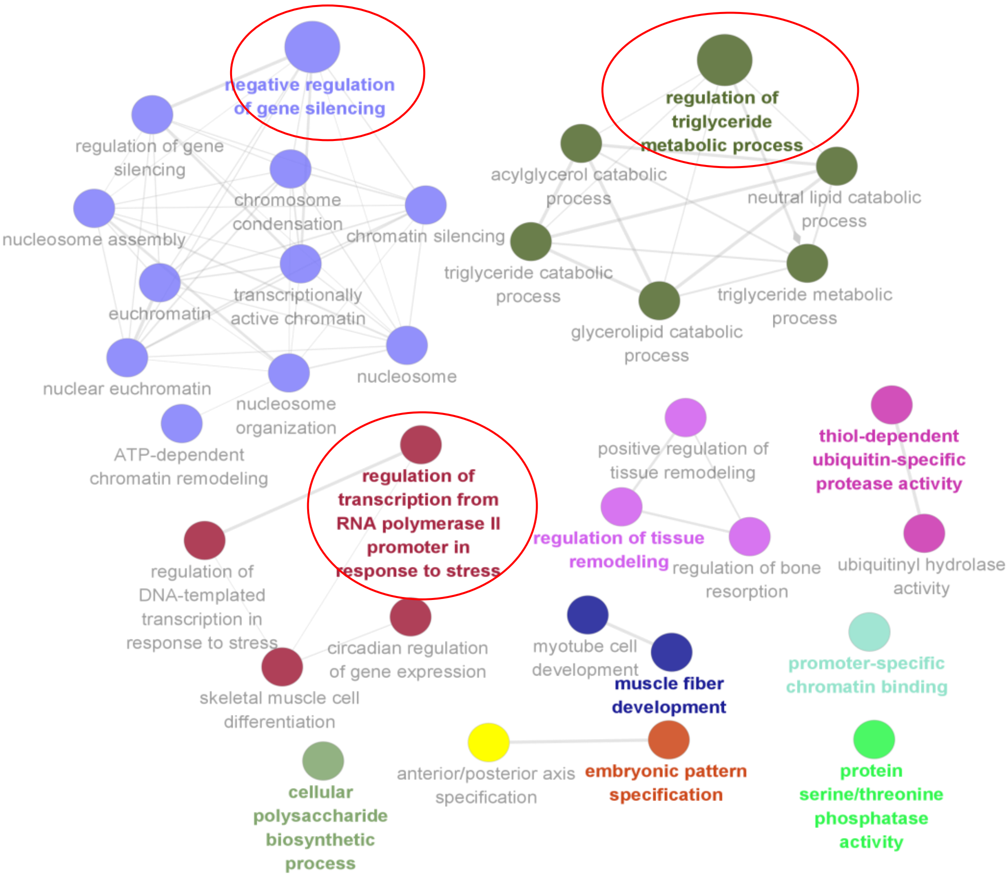

E L-AAPP vs M

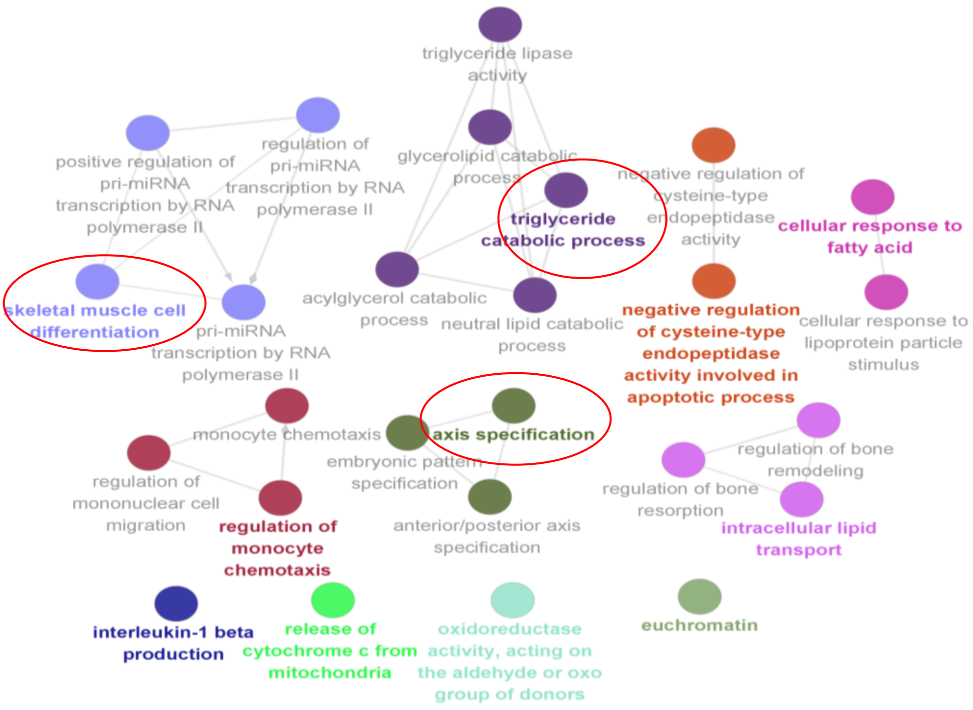

F H-AAPP vs M

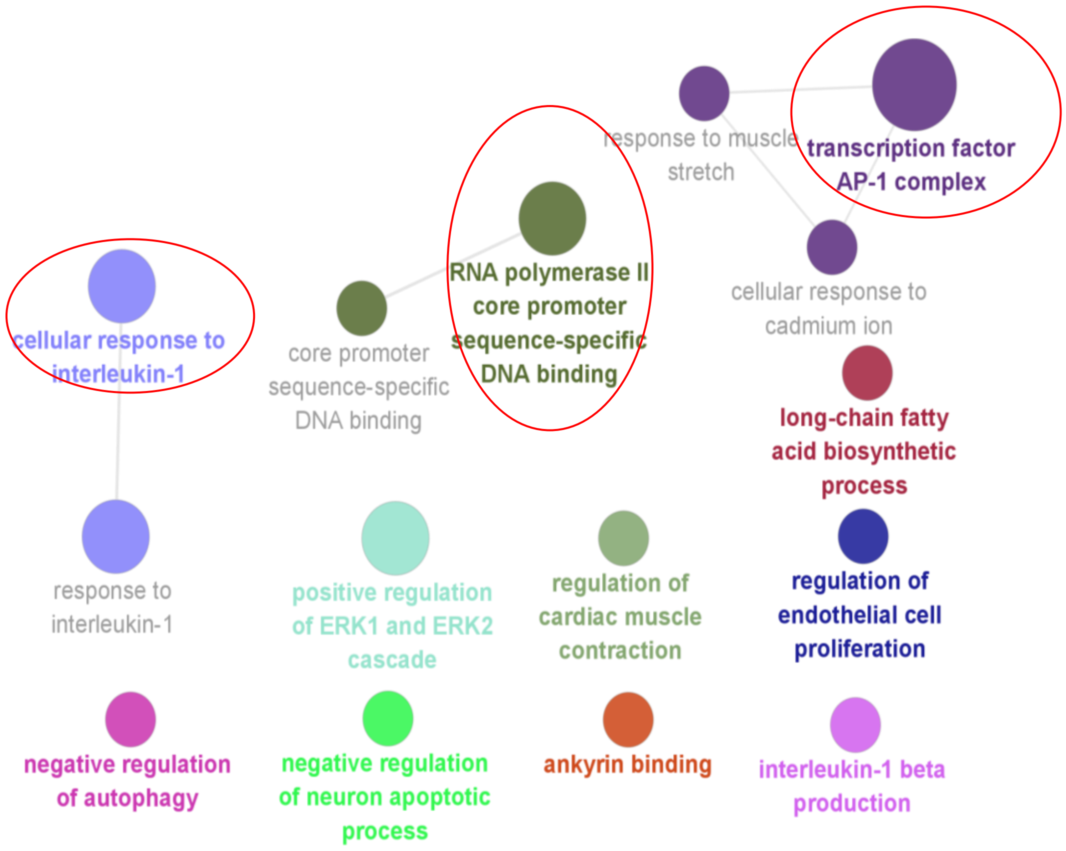

Figure S2. Functionally grouped networks of enriched GO terms were generated by the DEGs in Con/M (A), ND/M (B), L-NAAB /M (C), H-NAAB /M (D), L-AAPP/M (E), and H-AAPP/M (F) comparisons. The top 3 functional groups with the most abundant GO terms in each comparison are highlighted using red circles. GO terms are represented as nodes. The size of the nodes reflects the statistical significance of the terms and the color of the nodes reflects different functional groups. The nodes assigned with multiple colors indicate the nodes have multiple functions. H, high dose; L, low dose; NAAB, nonacylated anthocyanins extracted from bilberries; AAPP, acylated anthocyanins extracted from purple potatoes.

A

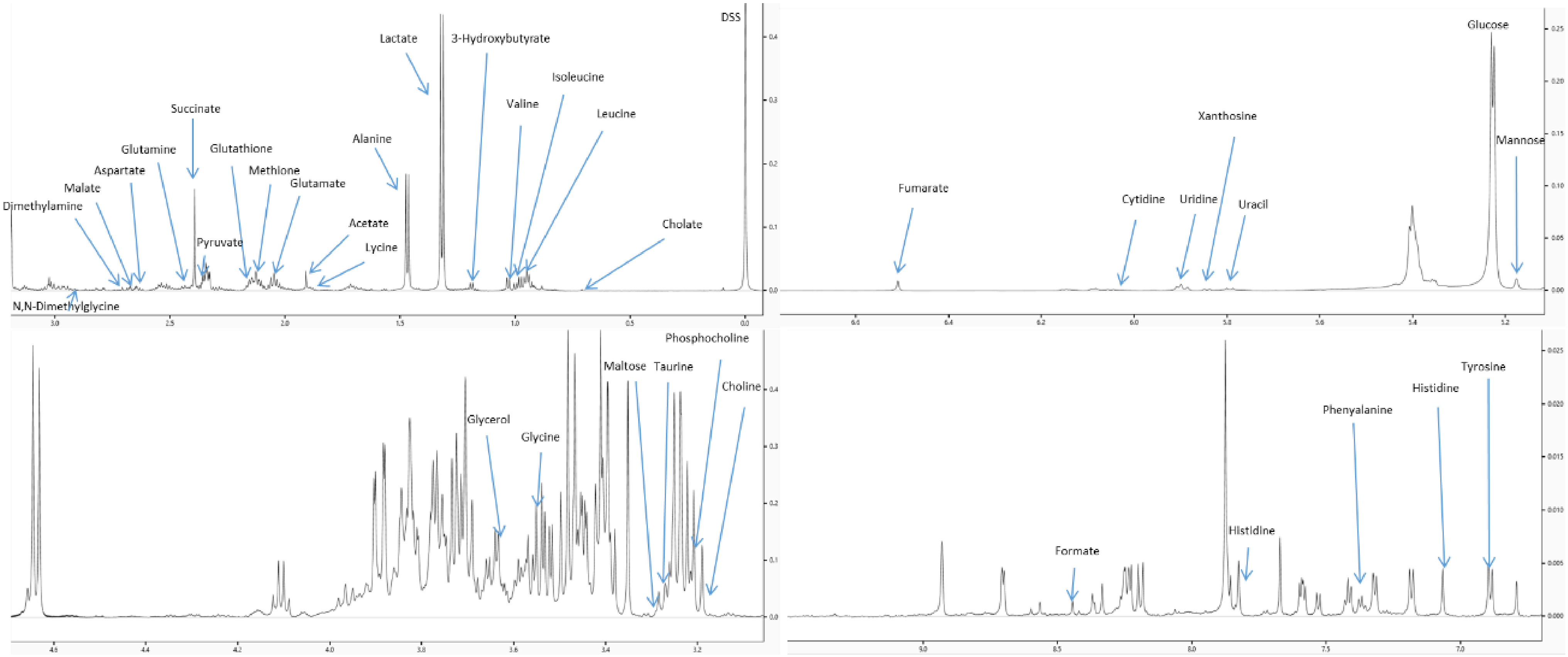
DSS
Lactate
3-Hydroxybutyrate
Isoleucine
Valine
Leucine
Succinate
Glutamine
Glutathione
Alanine
Aspartate
Methione
Glutamate
Malate
Dimethylamine
Pyruvate
Acetate
Lycine
Cholate
N,N-Dimethylglycine
Glucose
Xanthosine
Mannose
Fumarate
Cytidine
Uridine
Uracil
Phosphocholine
Maltose
Taurine
Choline
Glycerol
Glycine
Tyrosine
Histidine
Phenyalanine
Histidine
Formate

B

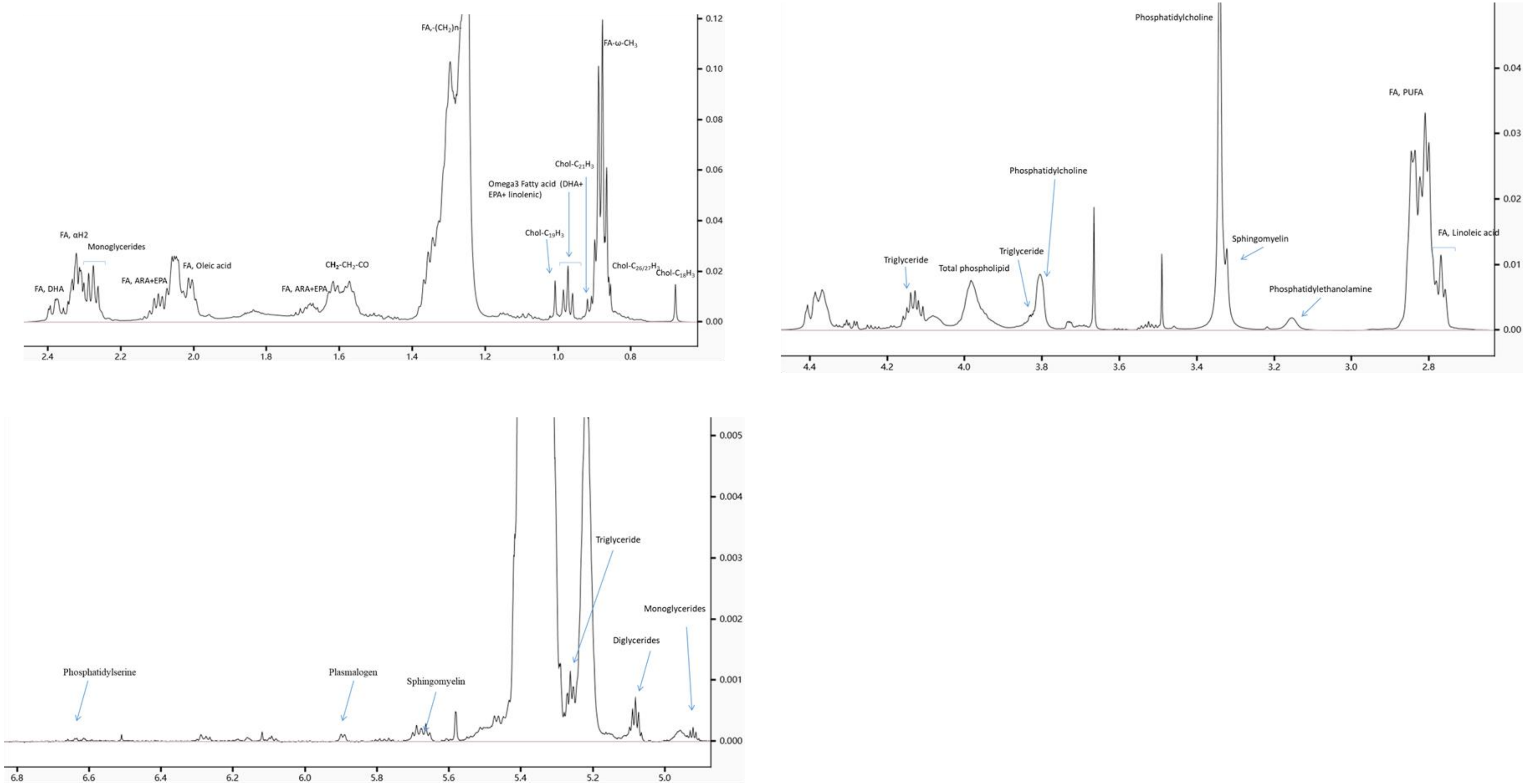

Figure S3. (A) 600 MHz noesypr1d ^{1}H NMR spectrum of aqueous extract from rat liver labeled with identified metabolites. (B) 600 MHz noesypr1d ^{1}H NMR spectrum of lipid extract from rat liver labeled with identified metabolites. FA, Fatty acid; DHA, docosahexaenoic acid; EPA, dcosapentaenoic acid; ARA, arachidonic acid; PUFA, polyunsaturated fatty acids.

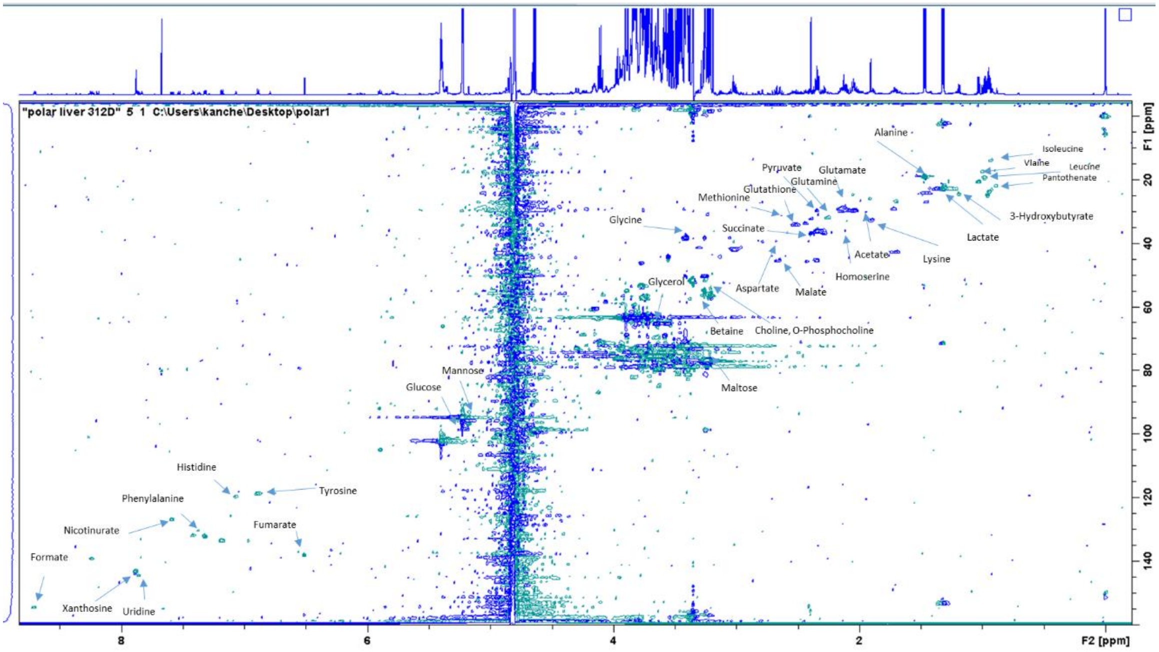

Figure S4 Identification of aqueous metabolites in rat liver in 2D NMR of HSQC

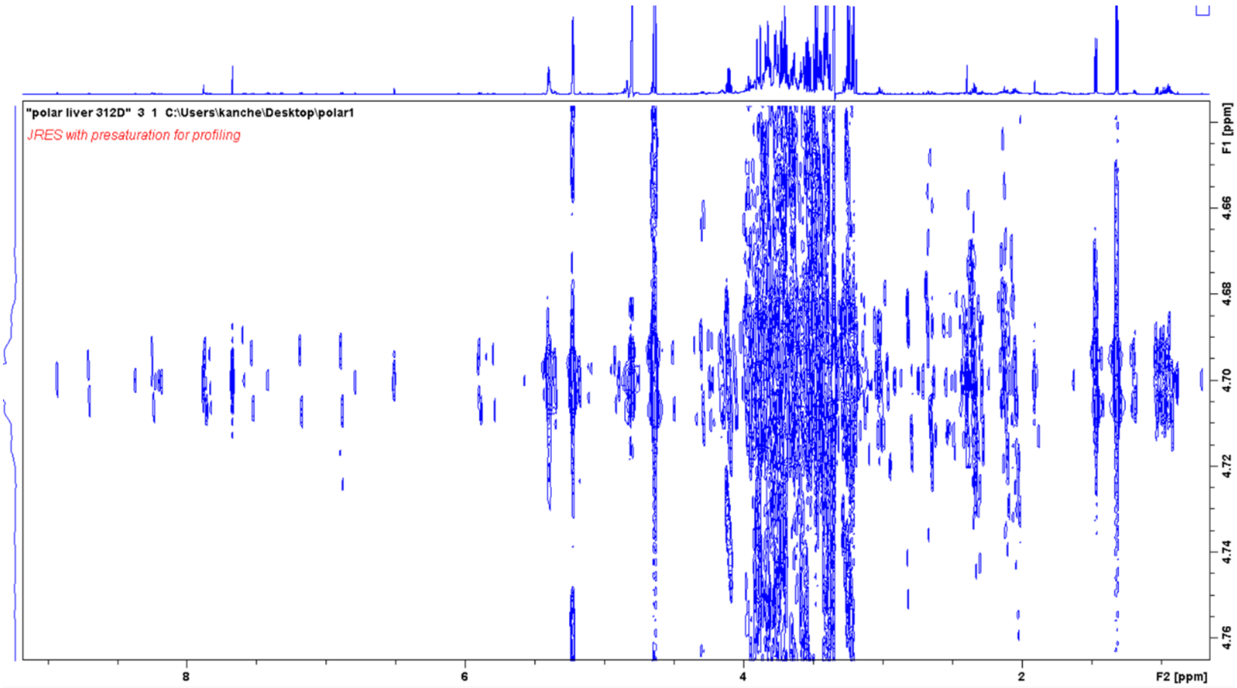

Figure S5. 2D JRES of aqueous metabolites in rat liver

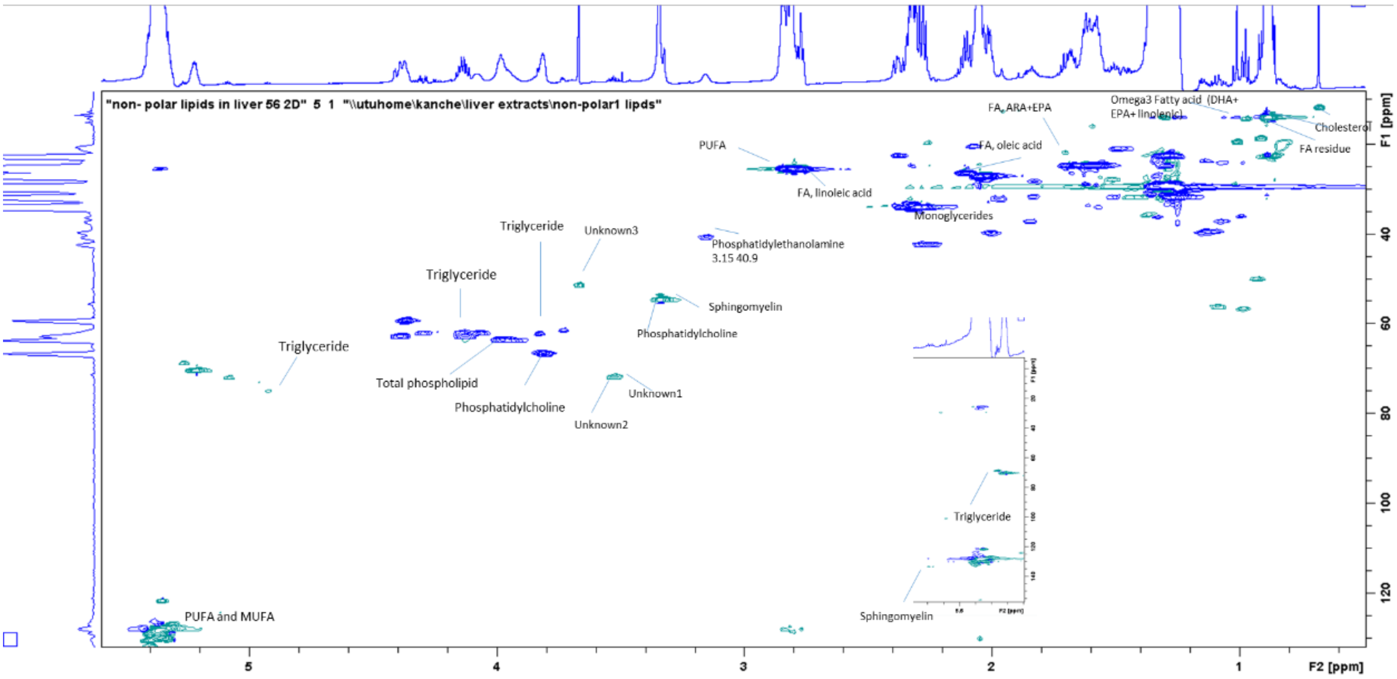

Figure S6 Identification of lipid metabolites in rat liver in 2D NMR of HSQC

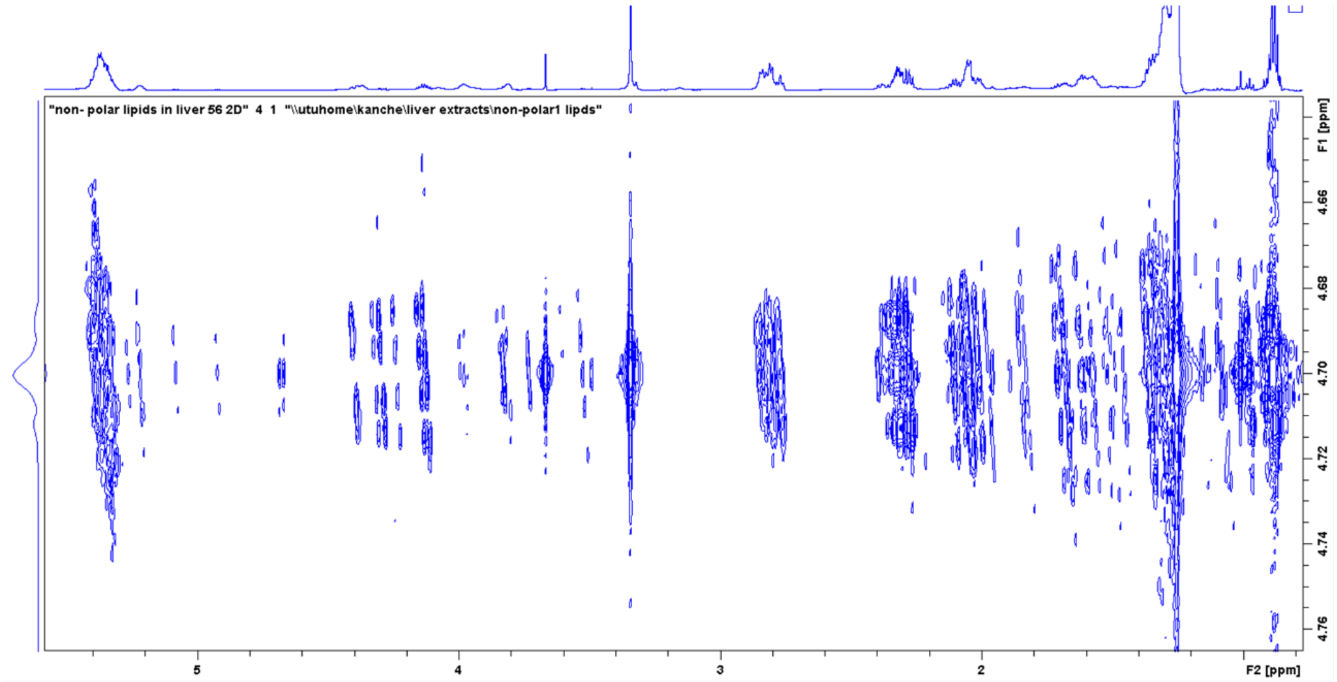

Figure S7. 2D JRES of lipid metabolites in rat liver.

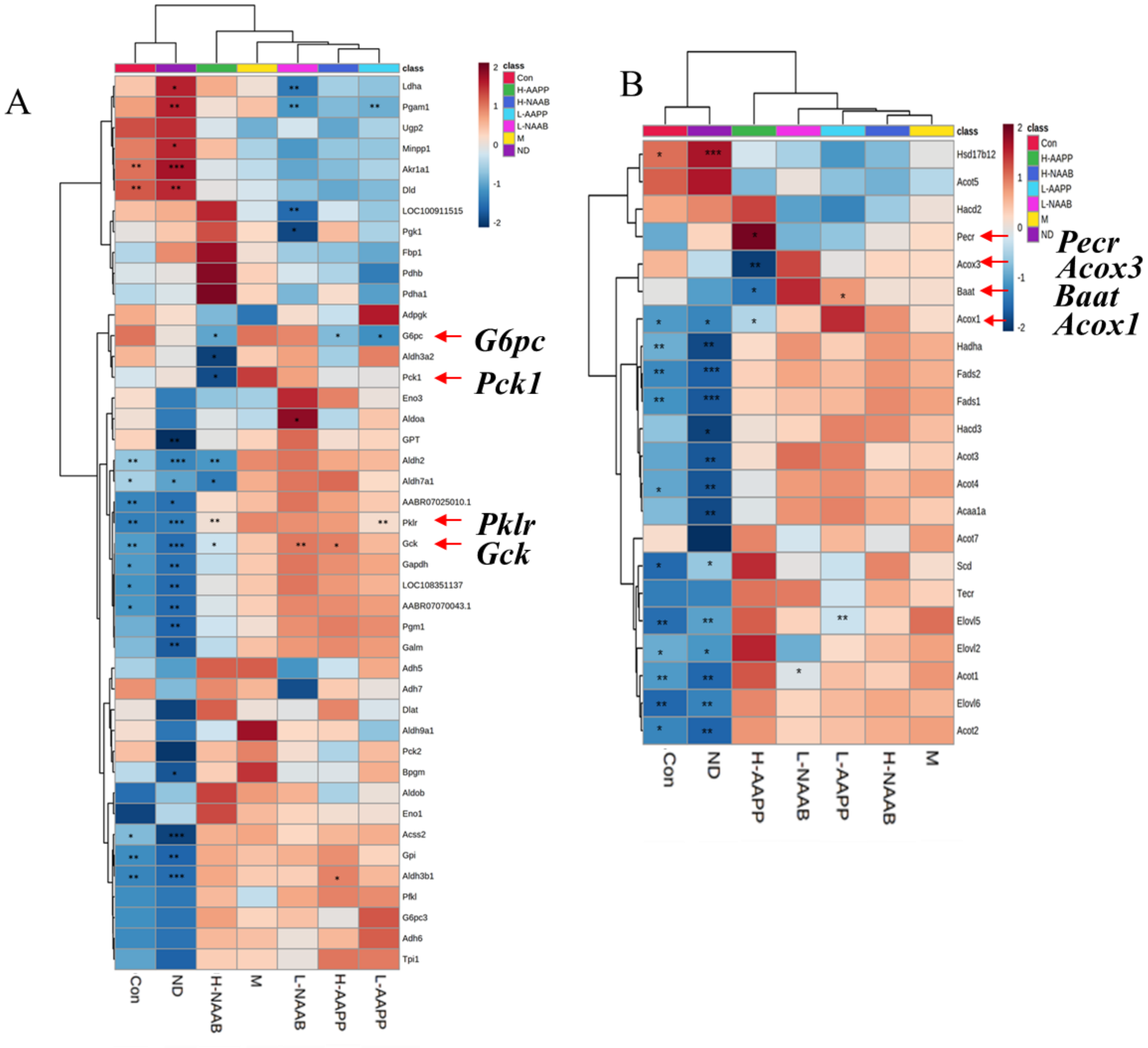

Figure S8. The heatmap of genes from Glycolysis/gluconeogenesis pathway from the KEGG pathway library (A). The heatmap of genes from Biosynthesis of unsaturated fatty acid pathway from the KEGG pathway library (B). *: p<0.05, **: p<0.01, ***: p<0.001 as compared with M group. H, high dose; L, low dose; NAAB, nonacylated anthocyanins extracted from bilberries; AAPP, acylated anthocyanins extracted from purple potatoes.

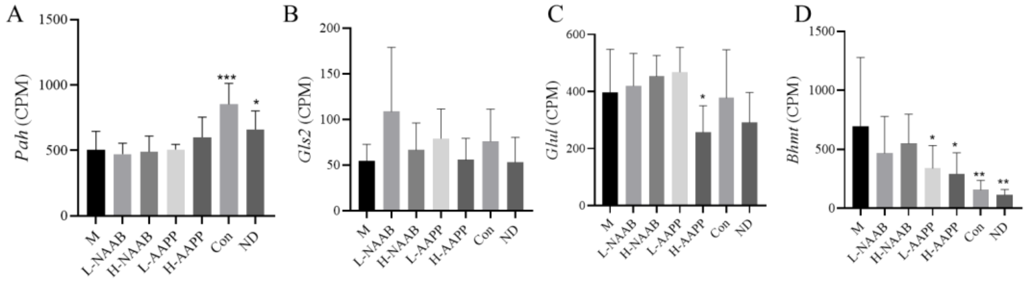

Figure S9 Hepatic mRNA expression for *Pah, Gls2, Glul,* and *Bhmt* detected in RNA-seq
